# Computational modeling of frequency-dependent neocortical response to thalamic neurostimulation in epilepsy

**DOI:** 10.1371/journal.pcbi.1012943

**Published:** 2025-04-28

**Authors:** Linda Iris Joseph Tomy, Elif Köksal-Ersöz, Anca Nica, Maxime Yochum, Pascal Benquet, Fabrice Wendling

**Affiliations:** 1 University of Rennes, Inserm-U1099, LTSI, Rennes, France; 2 Inria Lyon Research Center, Villeurbanne, France; 3 Cophy Team, Lyon Neuroscience Research Center, INSERM UMRS 1028, CNRS UMR 5292, Université Claude Bernard Lyon 1, Bron, France; 4 “Van Gogh” Epilepsy Surgery Unit, Neurology Department, CIC 1414, University Hospital, Rennes, France; Brandeis University, UNITED STATES OF AMERICA

## Abstract

The therapeutic application of centromedian nucleus stimulation (CMS) has been limited by uncertainties regarding its mechanism of action. In this study, we used stereoelectro-encephalography (SEEG) signals recorded from a patient with refractory epilepsy, caused by focal cortical dysplasia, which is a malformation of cortical development. SEEG recordings revealed that neocortical interictal discharges could be suppressed by CMS. These effects were found to be frequency-dependent: while 50 Hz CMS induced no change in neocortical epileptiform activity, CMS at 70 Hz, 100 Hz and 150 Hz led to periods of suppression of neocortical epileptiform activity. These periods were shown to have different durations depending on the stimulation protocol. We developed a neurophysiologically-plausible thalamocortical model to explain these observations. This model included glutamatergic subpopulations and GABAergic subpopulations in the neocortical and the thalamic compartments. Synaptic inhibition and short-term plasticity mechanisms were integrated into the latter compartment. We hypothesized that the enhanced activation of thalamic inhibitory subpopulations during high frequency CMS (>70Hz) would result in GABA spillover which activated synaptic GABAergic receptors on the thalamocortical relay cells. This decreased the thalamic driving-input to the neocortex, hence suppressing interictal discharges in the dysplastic neocortical tissue. While inhibition of thalamocortical relay cells was maximal for CMS at 70 Hz and 100 Hz, this was not the case for 150 Hz CMS, suggesting that presynaptic GABAergic receptors were activated and that the rate of GABA reuptake was increased. Thus, our model suggests that the transient suppression of the neocortical epileptic activity with CMS may be primarily due to extra-synaptic tonic inhibition in the thalamocortical relay cells. These findings contribute to a deeper understanding of high-frequency CMS in epilepsy and pave the way for further research and optimization of this therapeutic approach.

## Author summary

Focal cortical dysplasia is a malformation of cortical development. It is a common cause of drug-resistant epilepsy, characterized by recurrent seizures which strongly impact the patients’ quality of life. In these patients, exploratory stereo-electroencephalographic studies are performed to identify the epileptogenic zone(s) involved in the onset of seizures. Deep brain electrical stimulation has shown potential in management of ictal activity in such patients. The effect of stimulation is frequency dependent, but the underlying mechanism of action remains obscure, thus limiting its therapeutic efficacy. In this study we focus on the frequency-dependent suppression of interictal activity of the cerebral cortex in response to thalamic stimulation (namely, the centromedian nucleus of the thalamus). For this we employed a computational model, with which we modelled the patient’s intracranial recordings, and we decoded the mechanisms underlying these frequency-dependent effects: short-term synaptic plasticity in the cerebral cortex and extra-synaptic tonic GABAergic inhibition in the thalamus play a significant role in low- and high-frequency stimulation, respectively. Our results may be used to guide future therapeutic applications of this stimulation technique, thus encouraging its wider use.

## 1. Introduction

The centromedian nucleus (CMN) is an intralaminar nucleus of the thalamus, with reciprocal connections to premotor, motor, primary somatosensory and other neocortical regions. It is primarily involved in cognition, sensorimotor coordination and pain processing. Studies have evidenced the therapeutic effects of CMN stimulation (CMS) for epilepsy [1–6], Parkinson’s disease [7,8], focal cortical dysplasia (FCD) [9,10] and other neurological disorders. The stimulation of deeper cerebral structures is particularly important in refractory epilepsy [11–13]. In FCD, a type of neocortical developmental malformation that is commonly seen in epilepsy, the neocortical tissue becomes dystrophic while deeper brain regions, such as the thalamus, remain functional. As the thalamocortical pathway tightly controls neocortical activity [13–15], CMS is a potential therapeutic approach [6,16]. However, its application has been limited by a lack of understanding of the neuronal population-level dynamical mechanisms induced by CMS. It has been proposed that mid- to high-frequency CMS (70–150 Hz) may act through local excitation [17], local inhibition [18], or a combination of these mechanisms [19,20]. Some studies have also suggested the role of neuronal plasticity to explain their mode of action [21]. Given the complex nature of neuronal behavior, it may be a combination of these mechanisms that operate in a frequency-dependent manner. To date, modeling studies that implemented only one mechanism failed to simulate the frequency-dependent response elicited by deep brain stimulation (DBS) with a simplistic but neurophysiologically accurate model [17,21]. To address this limitation, we used neural mass modeling techniques to simulate StereoElectroEncephaloGraphy (SEEG) recordings obtained during CMS in a patient with FCD. Following the work reported in [21,22], we developed a novel thalamocortical computational model to explain the neocortical response to CMS. We improved the neocortical compartment by including vasoactive intestinal polypeptide-positive interneurons (VIP) and neuroglia form cells (Reelin-positive interneurons, INs; NGFC) subpopulations (proposed in [23]). Short-term plasticity (STP) mechanisms were also incorporated for the thalamic compartment and the glutamatergic thalamocortical connections [24–26]. As high frequency stimulation has been associated with increased activation of inhibitory synapses and the subsequent GABA spillover [27–32], our model included tonic extrasynaptic GABAergic inhibition to simulate this phenomenon.

Using this comprehensive model, we simulated neocortical and thalamic SEEG activity with close fidelity to the recorded SEEG. The patient’s SEEG signals showed frequency-dependent suppression of neocortical interictal discharges during CMS, with prolonged suppression following stimulation. We hypothesized that at high frequency CMS (≥70Hz), extrasynaptic receptors on the thalamocortical relay cells may be engaged by CMS-induced GABA spillover, which was not the case during 50 Hz CMS. Thus, in the absence of thalamic glutamatergic input to neocortical pyramidal cells and with the neuroplastic recovery of extrasynaptic tonic inhibition, it was possible to simulate the observed suppression of neocortical interictal discharges. We hypothesized that the activation of presynaptic GABA_B_ receptor (GABA_B_R) during 150Hz CMS would reduce the probability of release of GABA from thalamic INs, resulting in the rapid recovery of baseline interictal activity. Bifurcation analyses of the neocortical system confirmed that the interictal behavior of the neocortex was driven by the thalamic input.

In this study, we present a neural mass model of the thalamocortical network accounting for the immediate and the post-stimulation effects of CMS on neocortical interictal activity, with STP, presynaptic self-inhibition and extrasynaptic tonic inhibition mechanisms implemented in the model. The inferences obtained may be applied to plan effective therapeutic protocols in patients with focal refractory epilepsy.

## 2. Materials and methods

### 2.1. Ethics statement

The data used in this study were from a patient with drug-resistant epilepsy. This patient underwent a preoperative assessment using intracerebral electrodes to map the epileptogenic network. SEEG presurgical evaluation is a routine clinical practice in patient’s candidate to surgery intervention. It is approved by the legal Ethics Committee of the Rennes University Hospital (Comité d’Éthique du CHU de Rennes). The patient gave formal informed consent for the use of the collected data for research purposes, after anonymization. This formal consent was obtained verbally.

### 2.2. Clinical data

SEEG signals recorded from a 34-year-old right-handed man during presurgical SEEG exploration were used in this study. Based on his magnetic resonance imaging and electrophysiological data, the patient was diagnosed with refractory partial epilepsy due to FCD type IIB located in the premotor cortex [10].

**Fig 1:**
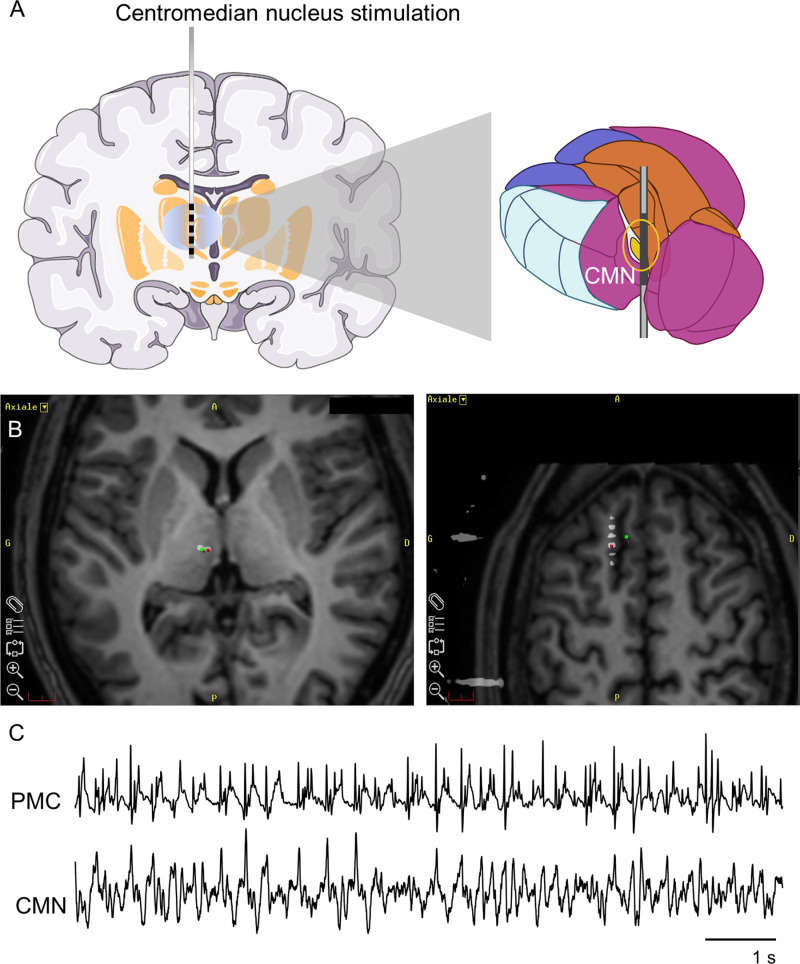
Centromedian nucleus stimulation (CMS) during StereoElectroEncephaloGraphic (SEEG) recording. (A) Schematic representation of a coronal brain slice showing a SEEG electrode used to stimulate the centromedian nucleus (CMN). Inset, the SEEG contact positioned near the CMN. (Drawn in part using images from Servier Medical Art) (B) Axial magnetic resonance imaging slices of the SEEG contacts positioned in the CMN of the thalamus (left panel), and in the premotor cortex (PMC; right panel). (Red: recording contact, green: adjacent contact). (C) SEEG recordings from the electrode contacts (in red, Fig 1B) positioned in the PMC and the CMN, in bipolar montage (with respect to the adjacent contact (green) in Fig 1B).

During the epileptogenic network mapping procedure performed in the Epilepsy surgery unit of the Rennes University Hospital, the CMN in the thalamus was stimulated, while the interictal activity was recorded from the dysplastic tissue (Fig 1A). Magnetic resonance images were acquired with a 3 Tesla 3D magnetic resonance imaging (MRI) scanner. A Leksell stereotactic frame and Medtronic StealthStation Neuro Navigation system were used to position the electrodes. Fig 1B depicts the positions of the CMN stimulation electrode and the neocortical electrode. SEEG was recorded from the premotor cortex and the CMN (Fig 1C). As depicted, the SEEG signal from the dysplastic tissue of the premotor cortex exhibited continuous interictal epileptiform activity. The timing of the negative peaks in the CMN recording coincided with the population spiking activity of the premotor cortex.

A five-second biphasic stimulation of 0.8 mA was applied at four specific frequencies (Table 1), using Dixi depth electrodes (6–18 contacts: 2 mm in length and 0.8 mm in diameter, 1.5 mm apart) and a Micromed video-SEEG recorder. Sustained suppression of neocortical interictal activity was observed at 70 Hz, 100 Hz, and 150 Hz stimulation, with 15-seconds of suppression at 100Hz stimulation. No suppression of neocortical interictal activity was observed with CMS at 50 Hz. The frequency-dependent suppression of interictal activity was found to be repeatable in this patient.

**Table 1:** Patient stimulation frequencies and response duration [10].

	Neocortical response				
CMS frequency	Before stimulation	During stimulation	2-seconds post-stimulation	15-seconds post-stimulation	30-seconds post-stimulation
50 Hz	–	–	–	–	–
70 Hz	–	✓	✓	–	–
100 Hz	–	✓	✓	✓	–
150 Hz	–	✓	✓	–	–

CMS: Centromedian nucleus stimulation; -: Absent; ✓: Present.

### 2.3. Thalamocortical model architecture

Neural mass models describe the average activity of neuronal populations. This approach was introduced by Wilson and Cowan [33], and the thalamocortical loop was added by Lopes da Silva [34]. These models have been used extensively to simulate the epileptiform activity [35, 36]. The general framework of the model presented here is based on [21,22].

This model consisted of two compartments (populations): a layered neocortical compartment and a thalamic compartment. Each compartment had excitatory and inhibitory subpopulations. These subpopulations were implemented according to the neural mass modeling formalism, where the average activity of each subpopulation is described by two main functions. The first one was a static sigmoid function (the “wave-to-pulse” function). It transforms the total postsynaptic potential (PSP) received by a subpopulation into a firing rate. The second one is a dynamic function (the “pulse-to-wave” function), transforming the firing rate into an average PSP with realistic kinetics (rise and decay time constants). This PSP can be either excitatory (EPSP) or inhibitory (IPSP) depending on the type of neurotransmitter released, typically glutamate or GABA, respectively. It is worth noting that two sub-populations of glutamatergic neurons are included to account for the recurrent (or collateral) excitation between pyramidal cells which is prominent in the neocortex, as described in [37]. Synaptic interactions among local neuronal subpopulations and between distant neuronal populations were represented by connectivity parameters.

**Fig 2:**
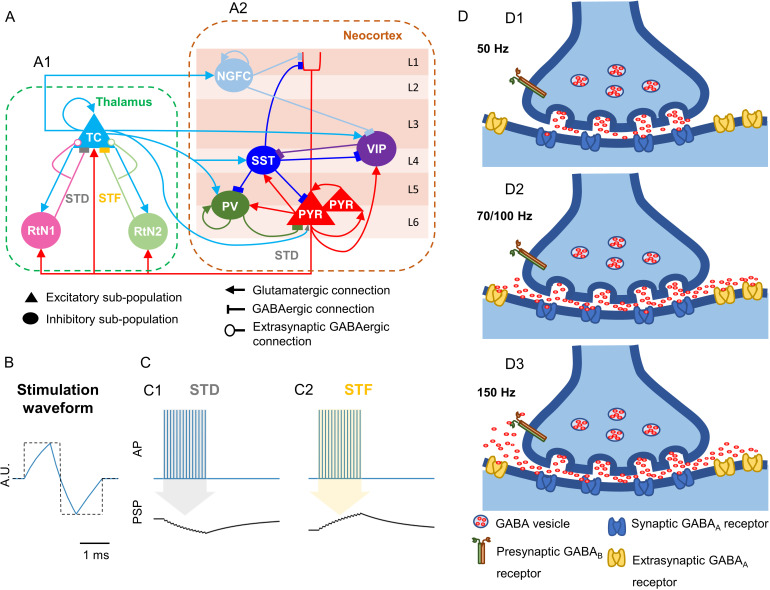
Thalamocortical neural mass model (NMM) and the mechanisms implemented in the model. (A) Schematic representation of the thalamocortical NMM. (A1) Thalamic compartment composed of an excitatory thalamocortical relay cells (TC) subpopulation and two inhibitory reticular nucleus (fast-kinetics: RtN1 and slow-kinetics: RtN2) subpopulations. (A2) Neocortical compartment composed of two pyramidal subpopulations (PYR), inhibitory somatostatin-positive (SST), parvalbumin-positive (PV), vasoactive intestinal polypeptide-positive (VIP) and neuroglia form cells (NGFC) interneuron (INs) subpopulations, with layer-wise distribution of these subpopulations. (L: Layer, STD: Short-term depression, STF: Short-term facilitation) (B) Distortion of the stimulation signal at the electrode-tissue interface. Stimulation waveform considered in the simulation studies, exemplified for 50 Hz. (In dotted black: a square biphasic pulse, in blue: stimulation waveform; A.U.: Arbitrary Units, ms: milliseconds) (C) Short-term plastic response to electrical pulse stimulation. (C1) STD: High frequency presynaptic action potentials (APs) train elicited by stimulation resulted in the depression of the post-synaptic potential (PSP) in the post-synaptic subpopulation. (C2) STF: High frequency presynaptic APs train resulted elicited by stimulation resulted in the facilitation of the PSP in the post-synaptic subpopulation. (D) Illustrations for the hypothesised neurological mechanisms occurring at RtN1/RtN2 synapses onto TC, for each stimulation frequency. (D1) 50 Hz stimulation: Typical GABA activity was engaged. (D2) 70/100 Hz: Higher level of GABA release resulted in GABA-spillover that activated the extra-synaptic GABA_A_ receptors. (D3) 150 Hz: Activation of the presynaptic GABA_B_ receptors reduced the probability of neurotransmitter release and increased the rate of reuptake.

The schematic representation of the model was as shown in Fig 2A. The neocortical excitatory subpopulations (PYR; $A_{c}$) represented the pyramidal neurons. The inhibitory subpopulations accounted for parvalbumin-positive (PV; $G_{c}$), somatostatin-positive (SST; $B_{c}^{i}$; i=a or b; a: apical pyramidal synapse, b: basal pyramidal synapse), VIP ($D_{c}\;$) and NGFC ($N_{c}$) INs. The PYR had direct excitatory connections to the PV, SST and VIP. In turn, the inhibitory subpopulations- SST, PV and NGFC- made direct inhibitory connections to the PYR. The inhibition loop between VIP and SST was also represented in the model [22,38]. The SST, VIP and NGFC make disinhibitory connections, inhibiting inhibitory subpopulations [23].

In the thalamic compartment, the thalamocortical relay cells of the CMN were represented by an excitatory subpopulation (TC; $A_{Th}$) [39,40], the reticular nucleus INs were represented by a fast-kinetics subpopulation (RtN1; $G_{Th}$) and a slow-kinetics subpopulation (RtN2; $B_{Th}$) [41,42]. The TC made excitatory connections to RtN1, RtN2 and to itself, and the RtN1/2 made inhibitory connections to TC. This model had feedforward excitatory connections from TC to PYR and vice-versa. Moreover, it also included a dual feedforward inhibition (FFI) system: cortico-thalamic connections from PYR to RtN1 and RtN2, and thalamo-cortical connections from TC to PV, SST and NGFC [38,43]. FFI allows for di-synaptic inhibition, which increases the threshold for activation and shortens the activation period of the targeted subpopulations [44,45].

To tune the parameter set of the thalamic compartment, we conducted a literature review to utilize physiologically realistic time constants for the subpopulations considered. The synaptic gains and connectivity constants of the model were manually tuned to produce simulated neocortical and thalamic SEEG signals that showed good agreement with the recorded SEEG signals in terms of morphological features and frequency, following both visual and computational validation (refer to Appendices A and N in S1 Text).

We have applied corticothalamic and thalamocortical delays based on [46,47]. To approximate the local field potential recorded at the SEEG electrode contacts, two monopoles in opposite directions were considered to account for current sinks and sources that occur in response to synaptic activation of PYR, either at basal or apical dendrites.

Numerical bifurcation analysis of the neocortical system was performed using XPPAUT [48]. Further details regarding the model architecture and implementation are provided in Appendix A in S1 Text.

### 2.3. Incorporating stimulation effects

The “λE-model” [49] was used in simulation studies to realistically model CMS. The “λE-model” ($\Delta V\approx \vec{\lambda }\cdot \vec{E}$) describes the linear relationship between the amount of neuron membrane polarization (ΔV), the membrane space constant $\left(\vec{\lambda }\right),$ and the magnitude/orientation of the electric field ($\vec{E}$; with respect to the main axis of neurons), induced by the electrical stimulation at the two adjacent contacts of the SEEG electrode in the CMN.

Following [21], different stimulation coupling constants were applied to each of the thalamic subpopulations (see Table A in S1 Text), to account for the variation in the magnitude of the electrical field within the CMN (where the SEEG electrode was located) and the reticular nucleus (which is further from the stimulation site).

The stimulation waveform was a 5 second (s) train of biphasic spikes (Fig 2B) at different frequencies (Table 1). The rise time constant was 0.001 s, the decay time constant was 0.0048 s, the up/down time was 0.001 s, the pulse width was set to 0.0005 s, and the amplitude was 3.2 arbitrary units. The parameterization was set to also account for the distortion of the stimulation waveform at the electrode-tissue interface which behaves like a RC-circuit. This stimulation waveform was applied to each of the thalamic subpopulations with weights that were appropriately tuned (refer Table A in S1 Text).

### 2.4. Short-term plasticity

Three connections were adapted to account for STP: TC to PYR expressed short-term depression (STD) [25,26], RtN1 to TC expressed STD, and RtN2 to TC expressed short-term facilitation (STF) [24]. This modulation in synaptic efficacy was modelled by varying the corresponding gain factors, $A_{C}^{d}$, $G_{Th}$ and $B_{Th}$, at the level of the targeted subpopulations (PYR, RtN1 and RtN2 subpopulations, respectively). The modulation was controlled by the firing rate of the efferent subpopulation [50,51], as follows:


$$\frac{du}{dt}=\left(\frac{u_{e}-u}{\tau_{f}}\right)+(1-uu_{e}\;fr\;stim$$ (1)

$$\frac{dk}{dt}=\left(\frac{1-k}{\tau_{d}}\right)-u\;k\;fr\;stim\;$$ (2)

$$x(t)=\frac{u(t)}{u_{e}}\;k(tx(t=0)$$ (3)

for $x\;\in \left\{A_{C},{G_{Th}\;,\;B}_{Th}\;\right\},$ variable k(t) is the amount of available resources (neurotransmitters; k(0)=1, 0<k<1), u(t) is the fraction of resources used by each presynaptic firing, $u_{e}$ is the baseline value of u(t), fr is the firing rate, stim is the stimulation variable, $\tau_{d}$ is the depression time constant, and $\tau_{f}$ is the facilitation time constant. The variables u(t) and k(t) were computed based on the firing rate of the presynaptic subpopulation using equations (1) and (2). During CMS (stim=1), the increased firing rate results in variations in u(t) and k(t)*.* In the absence of CMS (stim=0), these variables recover to their original values as the second part of the equations become zero. The gain factors were then computed by (3)..

### 2.5. Extrasynaptic tonic inhibition

During high frequency CMS, the inhibitory synaptic terminals on TC would be strongly activated [32]. Tonic inhibition has been shown to modify the activity of TC [52], and strong repetitive stimulation can activate such inhibition [29]. Thus, here we assume the synchronized firing of the thalamic inhibitory subpopulations. The resulting GABAergic spillover into the extrasynaptic space at the TC [31] would then activate extrasynaptic GABA_A_ receptors (GABA_A_R) giving rise to slow IPSPs in TC [29], as shown in Fig 2D1 and 2D2. We modeled this phenomenon for the connection between RtN1/2 to TC as follows,


$$\dot{y_{11}}=y_{24}$$

$$\dot{y_{24}}=\;E_{T}e_{T}y_{26}-2e_{T}y_{24}-e_{T}^{2}y_{11}$$

where$,\;y_{26}$ and $y_{11}$ represent the input for the sigmoid function and output from the dynamic function for the extrasynaptic GABAergic inhibition. The variable $y_{24}$ represents the first order differentiation of $y_{11}$. $E_{T}$ was the gain factor of extrasynaptic tonic IPSP and $e_{T}$ was the associated time constant.

The kinetics of GABA accumulation in the extrasynaptic space ($y_{26}$), was modelled as follows:


$${\dot{y}}_{26}=\;\left\{ \begin{array}{c} \;\frac{-y_{26}}{\tau_{IE}}+\phi_{RtN},\;with\;CMS\;(4)\\ \frac{-y_{26}}{\tau_{IE}}\;,\;without\;CMS\;(5) \end{array}\right.\;$$

$${where,\;\phi }_{RtN}=(\frac{G_{D}}{G}FR_{G}+\frac{B_{F}}{B}FR_{B}\backslash$$ (6)

where, $\tau_{IE}$ was the time constant for GABA reuptake. Equation (4) was formulated to simulate the consequent synchronous activation of extrasynaptic GABA_A_R which are activated by GABA spillover. In the thalamus GABA transporter-1 and GABA transporter-3 are responsible for GABA reuptake [53,54]. This activity has been simulated with (5). The combined firing rate of both slow- and fast-kinetics inhibitory subpopulations was given by the control parameter, $\phi_{RtN}$, computed using (6) [55]. This approach to model extrasynaptic inhibition is one of the novelties in this study.

### 2.6. Self-inhibition by presynaptic GABA_B_R

During 150 Hz CMS, we hypothesized that the spontaneous and rapid increase in GABA concentration around the presynaptic membrane would engage presynaptic GABA_B_Rs [56,57]. In this scenario, the GABA spillover mechanism gets arrested during the stimulation itself, as shown in Fig 2D3. We modelled this by increasing the $u_{e}$ parameter of RtN1 and RtN2 to 50 times its original values to simulate the reduction of presynaptic GABA release probability (as $u_{e}$ increases, the amount of resources utilized by each spike increases, thus limiting the presynaptic resource availability). The increased reuptake of GABA was also modelled by a decrease in $\tau_{IE}$ for GABA accumulation. This setting was engaged when GABA accumulation ($y_{26}$) exceeded a threshold value. The threshold was determined based on GABA accumulation observed during the 100 Hz-CMS simulation study (Appendix B in S1 Text).

## 3. Results

### 3.1. Signal comparison of neocortical and thalamic activity without stimulation: Recorded vs. simulated signals

**Fig 3:**
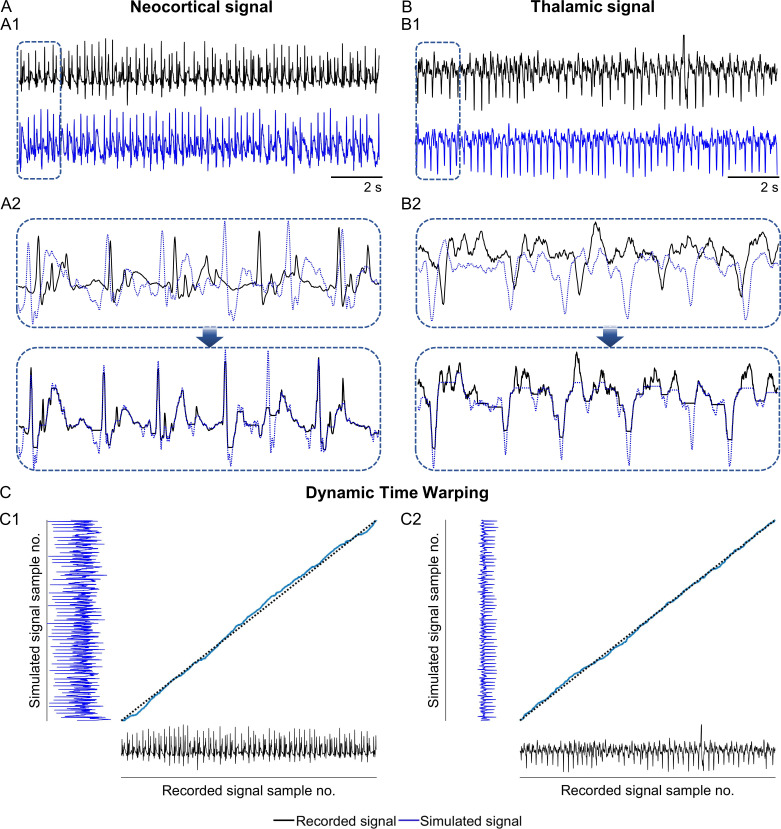
Comparison of the recorded and the simulated signals during interictal activity. (A) Neocortical signal (A1) Time domain plots of the recorded neocortical signal (black) and the simulated neocortical signal (blue), for a period of 14 seconds (s). (A2) Dynamically time warped simulated neocortical signal overlaid with the recorded neocortical signal, for a segment of ~1.5 s. (B) Thalamic signal (B1) Time domain plots of the recorded thalamic signal and the simulated thalamic signal, for a period of 14 s. (B2) Dynamically time warped simulated thalamic signal overlaid with the corresponding recorded thalamic signal, for a segment of ~1.5 s. (C) Dynamic time warping (C1) Time warping distance matrix for the neocortical signals. (C2) Time warping distance matrix for the thalamic signals.

The recorded neocortical SEEG showed interictal activity: ~3–4Hz periodic complex, composed of a fast spike followed by a slow wave (Fig 3A1). The recorded thalamic SEEG showed a spike-like behavior (Fig 3B1). The simulated neocortical SEEG and thalamic SEEG signals reproduced these waveforms, after manual optimization of the model parameters for connectivity and synaptic gain.

The similarity between the recorded and the simulated SEEG signals was assessed using dynamic time warping [58]. It was observed that the simulated signal closely resembled the recorded signal in terms of waveform morphology and frequency, (Fig 3A2, Fig 3B2). These depict a ~1.5 s (640 sample points) window of the dynamically time-warped simulated neocortical and dynamically time warped simulated thalamic signals overlaid with the corresponding dynamically time-warped recorded signals. Dynamic time warping was performed on a 14 s long recording to infer a realistic notion of the similarity between the signals (Fig 3C1-2). The optimal warping path showed minimal deviation from the diagonal, indicating close fidelity between the recorded and the simulated signals. The dynamic time warping score computed for the thalamic signals was 0.9920, and for the neocortical signals was 0.9904 (where 0 = no similarity and 1 = same signal).

### 3.2. Stimulation frequency-based response

Here we evaluate the congruence between the simulated and the recorded responses to CMS applied for 5 s (from 5–10 s). Only the stimulation frequency was changed for each simulation study, except for CMS at 150 Hz, where additional parameter changes were necessary (refer Section 2.6).

**Fig 4:**
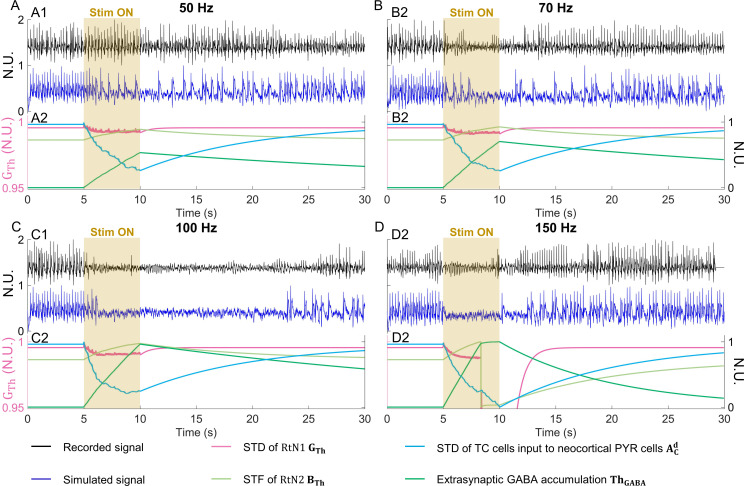
Recorded and simulated signals under centromedian nucleus stimulation (CMS), and evolution of dynamic parameters of the model. (A) 50Hz CMS (A1) No suppression of interictal activity in the recorded neocortical signal and in the simulated neocortical signal. (Stim ON: CMS period, N.U.: Normalized Units) (A2) Plastic parameter $G_{Th}$* depressed to 0.9921, $B_{Th}$ peaked at 0.8871, $Th_{GABA}\;$peaked at 0.5333, and $A_{C}^{d}$ depressed to 0.2601, at 10 seconds (s), and then recovered. (B) 70Hz CMS (B1) Suppression of interictal activity was observed during CMS in the recorded neocortical signal and in the simulated neocortical signal, followed by a short plastic suppression of ~2 s. (B2) Plastic parameter $G_{Th}$* depressed to 0.9913, $B_{Th}\;$peaked at 0.9245, $Th_{GABA}\;$peaked at 0.7021, and $A_{C}^{d}$ depressed to 0.2553, at 10 s, and then recovered. (C) 100Hz CMS (C1) Suppression of interictal activity was observed during CMS in the recorded neocortical signal and in the simulated neocortical signal. A 15 s plastic suppression period was observed and simulated, following CMS. (C2) Plastic parameter $G_{Th}$* depressed to 0.9906, $B_{Th}\;$peaked at 0.9753, $Th_{GABA}\;$peaked at 0.9636, and $A_{C}^{d}$ depressed to 0.2414, at 10 s, and then recovered. (D) 150Hz CMS (D1) Suppression of interictal activity was observed during CMS in the recorded neocortical signal and in the simulated neocortical signal, followed by a short plastic suppression of ~2 s. (D2) The plastic parameters $G_{Th}$* became 0, $B_{Th}\;$became 0, $Th_{GABA}\;$became 0.9760 and $A_{C}^{d}$ became 0.2517, at 8.35 s; but then $G_{Th}$* recovered to 0.6597, $B_{Th}\;$recovered to 0.0302, $Th_{GABA}\;$peaked to 0.9998, and$\;A_{C}^{d}$ evolved to 0.0142, at 10 s. *: Plotted on a separate y-axis with a different scale.

During 50 Hz CMS, the recorded neocortical signal showed no suppression of interictal activity during and after stimulation (Fig 4A). Similarly, the simulated signal also showed interictal activity during and after stimulation, albeit reduced. During 70 Hz CMS, the recorded neocortical signal showed suppression of interictal activity from 6.8-12.2 s, with low-amplitude spiking activity during 9.4-11.2 s (Fig 4B). In the simulated signal, suppression was achieved between 7.5-11.4 s. During 100 Hz CMS, interictal activity in the recorded neocortical signal was suppressed from 5.9-25.9 s, with lower amplitude spiking activity appearing as early as 22.2 s (Fig 4C). The simulated neocortical output showed suppression of interictal activity from 6.5-22.9 s. During 150 Hz CMS, interictal activity in the recorded neocortical signal was suppressed from 5-11.9 s, with low amplitude spontaneous spiking activity in this period (Fig 4D). But the recovery to baseline activity was at 19.5 s. In the simulated neocortical signal, interictal activity was suppressed from 5.4 -10 s. Baseline interictal activity reappeared after 12.25 s. The suppression of interictal activity with stimulation could be re-simulated with a variation in suppression period ±2 s, depending on the input noise to the compartments (refer to Appendix C in S1 Text). The number of interictal spike counts (pre- and post-stimulation) for both the recorded and simulated signals varied for each simulation scenario, as shown in Fig N in S1 Text.

We also investigated the evolution of the neuroplastic parameters with respect to the applied CMS frequency (Fig 4). For CMS frequencies between 50–100 Hz, we observed an inverse dependence of $G_{Th}$ and $A_{C}^{d}$ to the stimulation frequency, and a proportional dependence of $B_{Th}$ and $Th_{GABA}$ to it (refer to Appendix D and Fig G in S1 Text,). Neurophysiologically, this indicates that STD of the GABAergic synapses from the fast-kinetics inhibitory subpopulation to the thalamic excitatory subpopulation decreases at higher frequency of CMS. Whereas the STF of the GABAergic synapses from the slow-kinetics inhibitory subpopulation to the thalamic excitatory subpopulation increases with an increase of CMS frequency. The extrasynaptic GABA accumulation also increases with an increase in CMS frequency. This was not the case for CMS at 150 Hz. At this frequency, $u_{e}$of RtN1/RtN2, and $\tau_{IE}$ for GABA accumulation were varied at 8.35 s. This resulted in an abrupt drop of the plastic parameters $G_{Th}$and $B_{Th}\;$to 0, after which it recovered (refer to Appendix E in S1 Text). This decrease in GABAergic input from RtN1/2 to TC was induced by activation of presynaptic GABA_B_ receptors, which affects the probability of neurotransmitter release. Thus, GABA accumulation remained almost constant as the inhibitory subpopulations’ activity declined. This may indicate a change in the stimulation frequency-dependent dynamics for CMS at 150 Hz, which may be dissimilar from that observed for 50–100 Hz of CMS.

#### 3.2.1. Postsynaptic potentials of thalamic subpopulations.

We examined the PSPs of the thalamic subpopulations in response to CMS. As described in Section 2.2, the dynamic function in neural mass models is used to compute the PSP of each subpopulation from its firing rate.

**Fig 5:**
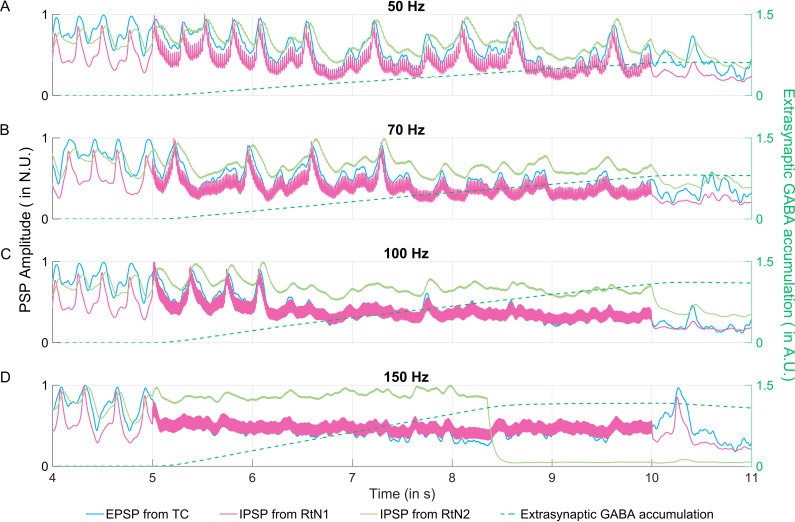
Simulated postsynaptic potentials (PSPs) of thalamic subpopulations. Simulated PSPs induced by thalamocortical relay cells subpopulation (TC), fast-kinetics inhibitory subpopulation (RtN1) and slow-kinetics inhibitory subpopulation (RtN2), during (A) 50 Hz, (B) 70 Hz, (C) 100 Hz and (D) 150 Hz centromedian nucleus stimulation, applied during 5-10 s.

The simulated PSP of each thalamic sub-population was as shown in Fig 5. In this model, the CMS amplitude was added to the PSP, and then input to the sigmoid function. Consequently, the PSPs simulated during CMS contained a high frequency component (corresponding to the frequency of the CMS signal). This was most prominently observed in the simulated IPSP from RtN1, likely owing to its higher time constants. The thalamic PSPs also contained a low-frequency component, 4 Hz, which was induced by the interictal activity from the neocortical compartment. The corresponding simulated LFP from the thalamic compartment was given in Appendix K in S1 Text.

During 50 Hz CMS, the PSPs remained mostly unaffected. As noted in the previous section, there was a decrease in the interictal spiking frequency, from 6.5 s of the simulated neocortical SEEG (shown in Fig 4A1). This also resulted in a decrease in the low frequency activity of the thalamic PSPs, from ~6.5 s, when $\begin{array}{c} Th_{GABA} \end{array}$ was 0.18 A.U. (Fig 5A). The activity of the thalamic inhibitory subpopulations resulted in the extrasynaptic GABA accumulation to 0.60 A.U. at 10 s. During 70 Hz CMS, the low frequency activity was reduced from 5.25 s, and disappeared at 7.3 s when $\begin{array}{c} Th_{GABA} \end{array}$ was 0.37 A.U. The continued activity of the thalamic inhibitory subpopulations resulted in an extrasynaptic GABA accumulation of 0.79 A.U. at 10 s. During 100 Hz CMS, the low frequency activity disappeared at 6.1 s, when $\begin{array}{c} Th_{GABA} \end{array}$ was 0.25 A.U. Extrasynaptic GABA accumulation then rose to 1.08 A.U. at 10s. During 150 Hz CMS, the disappearance of low frequency activity was at 5 s, when $\begin{array}{c} Th_{GABA} \end{array}$ was 0, but there was extrasynaptic GABA accumulation due to the CMS-induced activity in the thalamic compartment. This resulted in the extrasynaptic GABA accumulation being 1.16 A.U. at 10s.

### 3.3. Impact of mechanisms

We evaluated the impact of each of the STP mechanisms and the extra-synaptic tonic inhibition mechanism during 100 Hz CMS. At this stimulation frequency the suppression of neocortical interictal activity could be prominently observed. The variation in the number of interictal spikes was quantified from the simulated neocortical SEEG signals over 30 different noise initializations, for a variation in each of the implemented mechanisms. The same noise input was used in each of the 30 simulations.

**Fig 6:**
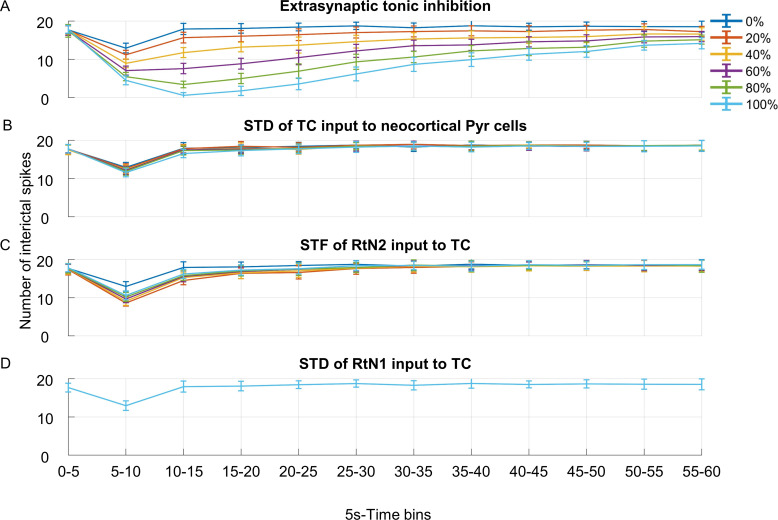
Impact of each mechanism on the number of interictal spikes. The number of interictal spikes in 5s-time bins for 60 s of simulated neocortical activity when the impact of (A) extrasynaptic tonic inhibition from the GABAergic connections to the thalamocortical relay cells subpopulation (TC), (B) short-term depression (STD) of input from TC to the neocortical pyramidal subpopulation (PYR), (C) short-term facilitation (STF) of the input from slow-kinetics subpopulation (RtN2) to TC, and (D) STD of the input from fast-kinetics subpopulation (RtN1) to TC was varied from 0 to 100%. Centromedian nucleus stimulation was applied at the 5-10 s time bin.

The effect of only implementing extra-synaptic tonic inhibition was evaluated by varying its synaptic gain ($E_{T}$) from 0–100% (0–0.125), as in Fig 6A. The effect of only implementing each of the STP mechanisms was evaluated individually by varying its baseline value $u_{e}$(the fraction of neurotransmitters used by each presynaptic firing), from 0 (when the STP mechanism was absent) to 100% of its $u_{e}$ value (given in Table A in S1 Text), as in Fig 6B-D. A change in $u_{e}$ affects the amplitude of the synaptic gain, according to equation (3) (Section 2.4).

Stimulation was applied at 5–10 s of the simulation time. Even without the STP mechanisms and/or extrasynaptic tonic inhibition, there was a decrease in the number of interictal spikes at the 5–10 s time bin, as seen from the plots when the impact of a mechanism was set to 0% (dark blue plots in Fig 6). On examining the PSPs of the thalamic compartment (Fig P in S1 Text) for this model configuration, it was seen that the relative increase in IPSP activity during CMS may have resulted in this decrease in the number of interictal spikes.

Increasing $E_{T}$ from 0 to 100% decreased the number of interictal spikes. The number of spikes was less than 5 during the 10-15s time bin for 80% (3.4±0.85) and 100% (0.57±0.73) of $E_{T}$. Such activity (1.73±1.2) could only be maintained for up to 15 s post-stimulation (20–25 s time bin) when $E_{T}$ was at 100% ($E_{T}$ = 0.125). Increasing $u_{e},$ from 20–100%, for the STD mechanism applied to the glutamatergic connection from the TC to PYR resulted in a decrease in the number of interictal spikes. This was particularly so at the 5–10 s time bin, from 12.6±1.25 to 11.57±1.14.

Increasing $u_{e}$ from 20–100%, for the STF mechanism applied to the GABAergic connection from RtN2 to TC subpopulation resulted in an increase in the number of interictal spikes. This was seen at the 5–10 s time bin, as the number of spikes increased from 8.6±0.83 to 10.6±1.16. Increasing $u_{e}$ from 20–100%, for the STD mechanism applied to the GABAergic connection from RtN1 to TC subpopulation did not seem to affect the number of interictal spikes.

When a plastic change in one of the dynamic parameters decreased the number of spikes during CMS, it subsequently increased the time for recovery to baseline-interictal behavior. None of the mechanisms by themselves could eliminate interictal activity completely (see also Appendices F and L in S1 Text). To accurately simulate the effect of 100 Hz stimulation we implemented extrasynaptic tonic inhibition for the GABAergic connections in addition to STD and STF at the level of thalamic compartment and STD for the glutamatergic connection of thalamocortical relay cells to neocortical pyramidal cells. For example, if thalamic extrasynaptic inhibition is removed the suppression period is reduced (see Table C, Fig I and Fig N in S1 Text). However, extrasynaptic inhibition alone is not sufficient, and STP mechanisms are also required to explain the observed post-stimulation effect. The different combinations of simulated mechanisms are as shown in Appendix F in S1 Text.

### 3.4. Bifurcation analysis

**Fig 7:**
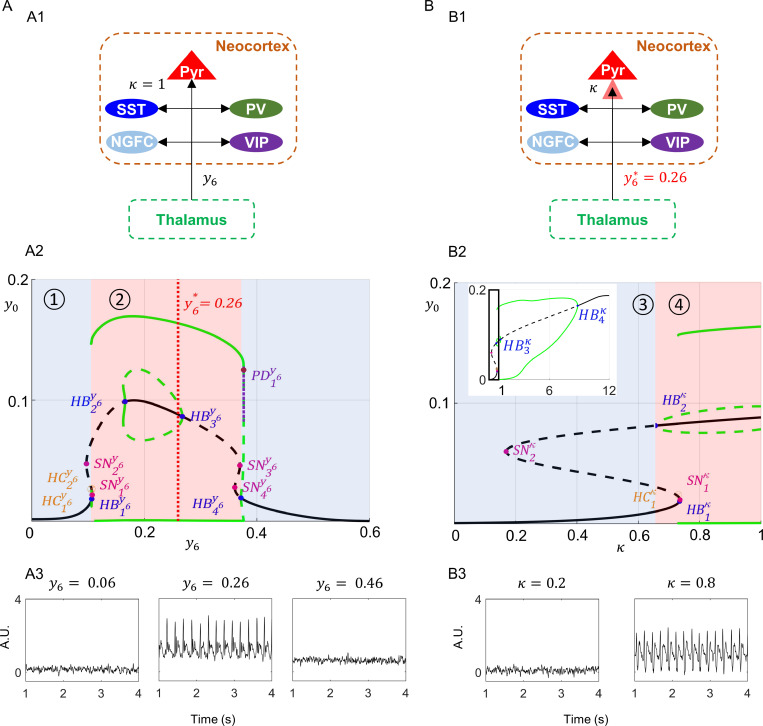
Bifurcation analysis of the neocortical pyramidal post-synaptic potential (PSP) as a function of the thalamic PSP. (A1) Schematic representation of thalamic PSP ($y_{6};\;$bifurcation parameter) applied to all the thalamocortically connected subpopulations of the neocortex, with the STD factor for thalamo-pyramidal connection, κ=1. (PYR: Pyramidal, SST: Somatostatin positive interneurons (INs), PV: parvalbumin positive INs, VIP: Vasoactive intestinal polypeptide positive INs, NGFC: Neuroglia form cell INs) (A2) Bifurcation diagram for neocortical pyramidal PSP ($y_{0}$) versus thalamic PSP ($y_{6}$). The black plots represent equilibria branches and the green plots represent periodic solutions, the solid lines depict branches of stable solutions and the dotted lines indicate branches of unstable solutions. The amplitude of periodic solutions of $y_{0}$ corresponded to the upper and lower branches of these solutions. The vertical line at $y_{6}^{*}=0.26$ marks the value used for the bifurcation analysis in Fig 7B. The graph had two monostable regions (shaded in blue) and a bistable region (shaded in red). As the input drive to the neocortex increased, the neocortical system transitioned from the monostable region, 1, to the bistable region, 2, on the right. ($HB_{i}^{j}$: Hopf bifurcation, ${SN}_{i}^{j}$: Saddle node bifurcation, $HC_{i}^{j}$: Homoclinic point, $PD_{i}^{j}\;$: Period doubling point; where i is the index and j is the bifurcation parameter) (A3) Simulated neocortical local field potential for $y_{6}=0.06,\;0.26$ and 0.46. (B1) Schematic representation of κ (bifurcation parameter) applied to the thalamo-pyramidal connection in the neocortex, with the thalamocortical PSP $y_{6}^{*}=0.26$. (B2) Bifurcation diagram for neocortical pyramidal PSP ($y_{0}$) versus STD factor (κ). Plot design and nomenclature corresponds to that followed in Fig 7A2. As the factor κ increased (thalamocortical drive increased), the neocortical system transitioned from the monostable region, 3, to the bistable region, 4, on the right. (B3) Simulated neocortical local field potential for κ=0.2 and 0.8.

We examined the nonlinear dynamical behavior of the neocortical system to variation in thalamic drive ($y_{6}$) (Fig 7A1; refer Table D in S1 Text), and its behavior to the variation of STD at the connection between TC to PYR by a factor κ (Fig 7B1; refer Table E in S1 Text), for the parameter set given in Table A in S1 Text. Note that both thalamic drive and the connectivity between TC to PYR are slowly changing quantities of the whole model, which led us to consider them as parameters for our analysis. This common approach inherits from the multiple timescale system theory [59].

We commenced the analysis for $y_{6}\in [0,0.6]$ by initializing κ=1 in the neocortical system (Fig 7A2). As $y_{6}$ increased from 0, the stable branch of equilibria became unstable at a subcritical Hopf bifurcation $HB_{1}^{y_{6}}$ for $y_{6}\approx 0.1067$. The periodic solution branch emerging from $HB_{1}^{y_{6}}$ made a homoclinic connection $HC_{1}^{y_{6}}$ at $y_{6}\approx 0.1062$. Following the branch of equilibria, the system underwent two saddle node bifurcations, $SN_{1}^{y_{6}}$ at $y_{6}\approx 0.1075$, and $SN_{2}^{y_{6}}$ at $y_{6}\approx \;0.0973$. The unstable branch of equilibria became stable between a supercritical Hopf bifurcation $HB_{2}^{y_{6}}$ at $y_{6}\approx 0.1657$, and a subcritical Hopf bifurcation $HB_{3}^{y_{6}}$ at $y_{6}\approx 0.2677$. We noted a bistable region of periodic and equilibria solutions between $HB_{2}^{y_{6}}$and $HB_{3}^{y_{6}}$*.*The periodic solutions branch connecting $HB_{2}^{y_{6}}$ and $HB_{3}^{y_{6}}$ were stable between $y_{6}\approx (0.1625,\;0.1657)$ and unstable $y_{6}\approx (0.1657,\;0.2677)$. Beyond $HB_{3}^{y_{6}}$ until $HB_{4}^{y_{6}}$ at $y_{6}\approx 0.3718$, the equilibria branch was unstable. We also observed two saddle node bifurcations, $SN_{3}^{y_{6}}$ and $SN_{4}^{y_{6}}$, at $y_{6}\approx 0.3700\;$ and $y_{6}\approx 0.3606$, respectively. The periodic solution branch that emerged from a subcritical Hopf bifurcation at $HB_{4}^{y_{6}}$ was initially unstable in $\;y_{6}\approx (0.3718,\;0.3767)$, and it was stable in $y_{6}\approx (0.1113,\;0.3768)$. A period doubling point, $PD_{1}^{y_{6}}$, appeared at $y_{6}\approx 0.3767$ (refer Appendix J in S1 Text). The limit cycles branch from $\;HB_{4}^{y_{6}}$ terminated at a homoclinic point $HC_{2}^{y_{6}}$ at $y_{6}\approx 0.01063$. The ~3–5 Hz interictal spiking behaviour corresponded to the stable periodic regime between $y_{6}\approx (0.1113,\;0.3768)$. This indicated that decreasing or increasing the thalamic input to the neocortical subpopulations can prevent interictal epileptic discharges.

Fig 7A3 shows the LFP dynamics of the modeled neocortical compartment for different values of $y_{6}$ corresponding to different regimes of the bifurcation diagram, in particular the monostable regime for $y_{6}\;=\;0.06$ and $\;y_{6}\;=\;0.46$, and bistable regime for $y_{6}\;=\;0.26\;$. When $y_{6}=0.26$ (region 2, Fig 7A2), the simulated system dynamics corresponded to interictal activity. When the thalamic input decreased during CMS, the system dynamics moved to $y_{6}=0.06$ (region 1, Fig 7A2) due to the activation of short-term facilitation of the RtN2-TC connection, which increases the GABAergic input on TC, and extrasynaptic tonic inhibition in the thalamic compartment, which decreases the thalamic activity globally. Thus, the implemented mechanisms suggested CMS induced the change in neocortical activity from epileptic discharges to normal.

From the bistable region 2, we chose to set $y_{6}^{*}=0.26$, as this corresponded to the average PSP from the TC to the neocortical subpopulations. With this setting, we performed a bifurcation analysis of the neocortical system as a function of the factor κ∈[0, 12] (Fig 7B) to investigate the impact of STD on the excitatory input from TC to the PYR. As the thalamic input (κ) increased, the system representing neocortical compartment underwent a subcritical $HB_{1}^{\kappa }\approx 0.7352\;$where the branch of equilibria became unstable. We observed two saddle node bifurcation $SN_{1}^{\kappa }$ and $SN_{2}^{\kappa }$ at κ≈0.7365 and κ≈0.1695, respectively. The equilibria branch was stable between the $HB_{2}^{\kappa }$ (κ≈0.6564) and $HB_{3}^{\kappa }\;(\kappa \approx 1.0904)$, with a branch of unstable periodic solutions emerging from $HB_{2}^{\kappa }$ and terminating at $HB_{3}^{\kappa }$. The unstable equilibria branch then continued until the supercritical Hopf bifurcation $HB_{4}^{\kappa }$ at κ≈8.8383, after which the equilibria branch was stable. A stable periodic solutions branch emerged from $HB_{4}^{\kappa }$ and $HC_{1}^{\kappa }$ at κ≈0.7307. We can see from the bifurcation diagram (Fig 7B2) that only the lower branch, κ=(0, 0.6564), corresponded to background-activity without interictal discharges.

Fig 7B3 shows the LFP dynamics of the neocortical compartment for κ=0.2, corresponding to the monostable regime (region 3, Fig 7B2), and κ=0.8, corresponding to the bistable regime (region 4, Fig 7B2). This indicates that weakening of the excitatory thalamic input via STD can be one of the mechanisms to suppress interictal activity in the neocortex. Note that this suppression is transient as STD corresponds to a transient decrease of the synaptic gain.

## 4. Discussion

In this study, we report that CMS suppressed neocortical interictal epileptiform activity in a frequency-dependent manner. While 50 Hz CMS had no effect, CMS at higher frequencies (70 Hz, 100 Hz and 150 Hz) produced variable periods of suppression. The proposed neurophysiologically plausible thalamocortical model suggests that high-frequency CMS may activate thalamic inhibitory subpopulations, leading to GABA spillover and extra-synaptic tonic inhibition of thalamocortical relay cells. This reduction in thalamic input to the neocortex explains suppression of interictal discharges. In addition, this study incorporates short-term plasticity mechanisms in the thalamocortical circuit. With this, the model suggests that activation of the extra-synaptic inhibition by CMS could be the main mechanism for the suppression of epileptic activity in the neocortex.

The thalamocortical model used in this study incorporates a layered neocortical compartment [23]. It includes the representation of VIP and NGFC in the neocortical compartment, unlike previous thalamocortical models [21,60,61], as these INs have been shown to receive strong inputs from the thalamus [38,62,63]. Although we did not investigate the impact of VIP and NGFC subpopulations on the epileptic activity or their role under CMS, we believe that the model stays relevant, not only in this particular study and but also for future work.

In addition, we observed that the PV-PV connection did not significantly determine the morphology of the interictal discharges, in contrast with the fast activity observed at the onset of focal seizures [64,65]. Therefore, the PV-PV connection value was set to zero, even though the connections are present in the model itself (refer Appendix N in S1 Text). Along the same line, we did not consider the PV-SST connection in the model because the connectivity probability is rather low compared to other inhibitory connections on the SST subpopulation in the neocortex [66].

We implemented a rudimentary model to represent the thalamus [21,34,67], we included a self-connection in the TC subpopulation to represent synapses from adjacent nuclei and CM nuclei onto the thalamocortical neurons. The corticothalamic and thalamocortical coupling constants were grossly based on previous [43,68–70]. In terms of model complexity, the proposed model is simple enough to be controllable, but not simplistic. It provides valuable insights into the dynamics of SEEG signals recorded in the neocortex for the different stimulation frequencies applied to the CMN. To begin with an even simpler model, would mean losing the neurophysiological likeness of the current approach.

Each thalamic nucleus is known to be closely associated with a population of INs in the thalamic reticular nucleus [71]. Thalamic slow and fast kinetics inhibitory subpopulations have been reported in studies conducted in rodents and humans [39,41,42]. For simplicity, we assumed that the influence of local INs of the CMN would be similar to that of the inhibitory fast-kinetics subpopulations (RtN1). We based the thalamic time constants on literature (refer Appendix A in S1 Text). Given the flexibility offered by neural mass modeling, we manually tuned synaptic gains and connectivity values to improve the congruence between the morphology and frequency of simulated signal to that of the recorded signal. Our thalamic model could not simulate low threshold spiking behavior, which is characteristic of thalamocortical relay cells. We assumed this behavior was inconsequential in frequency-dependent CMS response, hence we proceeded without it.

Simulation studies for DBS have evidenced the decay of electric potential away from the stimulation contacts, with stronger field strengths closer to the electrode contacts [8,72]. It was also observed that the thalamocortical relay cells predominantly receive excitatory synapses in the ventral posterior thalamic nuclei [84]. Based on these notions, we employed differential stimulation coupling coefficients to apply stimulation to the thalamic subpopulations, with a higher value for the TC subpopulation. Such an approach was also used in [21].

When comparing the simulated and recorded signals, a few inconsistencies could be noted in the simulated signal regarding the spiking behavior. But the recorded signal itself displayed varied dynamics over time (refer to Appendix H in S1 Text). Hence, we chose to replicate the prominent periodic complex and neglected the sporadic lower amplitude spiking behavior. The negative peaks in the thalamic recording induced by neocortical spiking (refer to Appendix H in S1 Text) were also reproduced in the simulated signals. The congruence between the simulated and recorded signals, and thus the accuracy of the model, was confirmed by using dynamic time warping algorithm, which is considered a reliable metric to estimate the similarity of patterns between the signals [73]. Indeed, during the pre-stimulus period, the SEEG recordings in the dysplastic neocortex showed typical epileptiform activity (high-amplitude rhythmic spikes) for which the dynamic time wrapping method was well suited to quantify the morphological similarity between the recorded and the simulated signals. Whereas, during/post-CMS, the SEEG activity returned to a normal state in which the interictal epileptic discharges activity was absent. In this case, dynamic time wrapping was not appropriate since the stimulation-induced activity was more random (Fig 4C).

In addition, regarding the suppression of spiking activity (post-stimulus) and regarding the time taken for the dynamics to return to pathological state, the model showed that these two features are directly related to (i) STD of TC cells input to neocortical pyramidal cells, (ii) STD of RtN1 cells, (iii) STF of RtN2, and (iv) extrasynaptic accumulation of GABA, as shown in Fig 4.

Given the complex nature of neuronal systems, the response elicited by CMS could likely result from the interplay between multiple mechanisms. Hence, in the present study we integrated physiological mechanisms, STP and extrasynaptic tonic inhibition, to simulate the neocortical response to CMS. The time scale of these mechanisms was comparable to that of the recorded neocortical responses to CMS, when considering the duration of stimulation.

### 4.1. Tonic inhibition in CMN

During CMS, the axonal terminals onto the TC (both excitatory and inhibitory) may be activated [8,72]. High frequency stimulation engages inhibitory populations more strongly than excitatory populations [32]. Also, extracellular stimulation of the thalamic nuclei increases local inhibitory currents [7,74]. This indicates that the inhibitory currents would be strongly impacted during CMS.

Normal synaptic inhibition is mediated by phasic currents and tonic currents. Thalamocortical relay cells exhibit tonic inhibition [29,52,75–77], which can be enhanced by stimulation [7,32,74]. GABAergic inhibition is mediated synaptically and extrasynaptically by GABA_A_Rs, and pre-synaptically by GABA_B_Rs [29,31,52,78]. In the thalamocortical relay cells, GABA_A_Rs get activated by synchronous activation or by repetitive stimulation [78,79]. When inhibitory neurons are selectively activated during high frequency stimulation (as described above), an influx of calcium ions occurs. This facilitates the release of “reserve pools” of GABA from these neurons (in addition to the “readily releasable pool”) [80,81], resulting in GABA spillover.

These results led us to hypothesize that during high frequency CMS, the inhibitory synapses onto the CMN may be strongly activated, which would cause the release of the “reserve pool” of neurotransmitters. The resulting GABA spillover [79] could then cause the activation of extrasynaptic GABA_A_Rs. Thus, we modelled activation of extrasynaptic GABA_A_Rs depending on GABA accumulation which controlled extrasynaptic tonic inhibition potential. In our formulation for GABA accumulation we followed the results from [55], such that it was directly proportional to the strength of phasic inhibition.

Strong extracellular activation has been shown to be necessary to activate postsynaptic GABA_B_Rs [79,82]. This showed that a concomitant release of GABA can activate these receptors as well. But as reported in [27], the effect of GABA_B_R activation only slightly modulates the effect of high frequency stimulation. This receptor has been observed to have an influence in long-term effects of stimulation [83–85]. But due to the lack of evidence regarding the influence of this receptor in response to stimulations applied for a short period, it has not been explicitly included in the current model.

In Lee et al. (2011), the authors suggested that high frequency stimulation leads to the hyperpolarization of thalamocortical relay cells, which is consistent with our model observations [17]. In their study, the hyperpolarization current was linked to glutamate release to simulate the effect of stimulation. In our work, GABA spillover seemed to be a possible outcome to CMS as thalamocortical relay cells are known to be tightly controlled by inhibitory inputs from the thalamic reticular nucleus. In McIntyre et al. (2004), the authors developed a detailed two-neuron model to simulate the neuronal response to the electric fields induced during deep brain stimulation [86]. They reported that GABA_B_ conductance was associated with the suppression of thalamic activity with 150 Hz stimulation. However, this two-neuron model could not explain frequency dependent response to deep brain stimulation.

The model presented in [21] also used the SEEG data from [10] to develop a computational model for simulating the response to low frequency CMS. They suggested that the activation of strong inhibitory currents in TC would suppress the thalamocortical drive to PYR. This notion was also consistent with what was observed in the present study.

### 4.2. CMS modeling inferences

We first assumed that four mechanisms were necessary to reproduce the observed effects of CMS on neocortical SEEG signals: STD at the thalamocortical relay cells subpopulation to the pyramidal subpopulation connection, STD at the fast-kinetics subpopulation to the thalamocortical relay cells subpopulation connection, STF at the slow-kinetics subpopulation to the thalamocortical relay cells subpopulation connection, and extrasynaptic tonic inhibition. We then implemented these four mechanisms in the model which offered the possibility to analyze each mechanism separately. The results showed that the initial assumption was valid and that the four considered mechanisms were necessary and sufficient to reproduce the observed frequency-dependent effects of CMS.

Based on the simulation results for 50 Hz CMS, we inferred that suppression of neocortical interictal discharges could only be achieved when the PSP of the thalamic subpopulations decreased. Among the mechanisms implemented in our model, extrasynaptic tonic inhibition was essential to observe continuous post-stimulation suppression of neocortical interictal activity. However, this mechanism by itself could not simulate the response observed during 100 Hz CMS. Either depression of TC synapses onto PYR, or facilitation of RtN2 synapses onto TC, was necessary to simulate the observed suppression. The activation of STP mechanisms by CMS were seen to be responsible for the immediate suppression of interictal activity. STP mechanisms also played a role in delaying the recovery of TC activity, hence prolonging the observed suppression. Thus, the STP mechanisms contributed to improving the agreement between the simulated and the recorded SEEG.

We also studied the response of the neocortical system to the thalamic driving-input using bifurcation analysis. This confirmed that a decrease in this driving input to the neocortical pyramidal subpopulation can indeed switch the system between interictal and background regime. A stochastic input (in the present study, from the thalamus), when the neocortical system is at the boundary between normal and interictal activity, could result in transitions between these regimes. This could be translated as variations in the suppression period. These inferences could be used in therapeutic applications of stimulation, to control either neocortical activity or the input drive from the thalamus to the epileptogenic zone.

Theoretically, tonic inhibition of TC could be achieved by increasing stimulation duration, increasing stimulation amplitude or by increasing stimulation frequency. If longer stimulation period were used, GABA diffusion away from the synaptic cleft would be highly likely. In Toprani et al. (2013), the authors employed low frequency stimulation for 15 minutes and observed more than 50% reduction in epileptic activity 15 minutes after stimulation, but they were unable to abolish epileptic activity [85]. GABA_B_Rs were also found to play an important role in this reduction.

In clinical practice, stimulation strengths are chosen to safely stimulate the tissue without causing permanent neuronal tissue damage. Thus, increasing the stimulation intensity is not recommended. Therefore, it seems that the optimal choice would be to increase the stimulation frequency. However, as evidenced in our results, this strategy has its own limitations, namely activation of auto receptors (as discussed below). Similar findings have also been observed in [7,87], where the stimulation loses its efficacy beyond a certain frequency.

### 4.3. Why not at 150 Hz?

The frequency dependent response of neuronal circuits to stimulation has been observed in several *in vitro* [87] and *in vivo* studies [7,11,74]. But the mechanism involved in eliciting this selective response remains unclear. Tobochnik et al. (2024) reported a case study where *status epilepticus* could be terminated with CMS at 100 Hz [3]. And from Lee et al. (2003), subthalamic nucleus stimulation was shown to induce maximal firing only for stimulation frequencies in the range of 100–140Hz [87]. This was also the case in the current patient. Optimal response was only seen to increase from 70 Hz to 100 Hz, and then was almost absent at 150 Hz CMS, post-stimulation. Here we suggested the causative role of pre-synaptic GABA_B_Rs to explain this behavior [78,88,89].

During 150 Hz CMS, the rate of GABA neurotransmitter release by the thalamic inhibitory neurons would be higher than at 100 Hz, due to the higher CMS-driven PSPs induced during 150 Hz CMS. In this scenario, the GABA released may accumulate near the presynaptic site. This presynaptically accumulated GABA would then activate the presynaptic GABA_B_Rs associated with the thalamic inhibitory subpopulations, which would reduce the GABA release probability of those INs [57]. Hence, the presynaptic accumulation of GABA during 150Hz stimulation may be the reason for the observation of an optimal frequency range in this CMS dataset.

As reported in Gonzalez-Burgos et al. (2009), GABA transporters play a role in limiting GABA spillover in neocortical pyramidal neurons [90]. In addition, GABA transporters-1/3 in the thalamus have been reported to modulate phasic and tonic GABA release, respectively [53]. Thus, we implemented an increase in the rate of GABA reuptake. In the absence of this premise in our model, the CMS-induced neocortical interictal suppression would be longer than 15 s (refer to Appendix I in S1 Text), which would be inconsistent with the SEEG observations from the patient. Regarding presynaptic GABA transporters activation during GABA spillover, it is worth noting that several studies show that it exists in cortex [90] and the hippocampus [91]. As these transporters are also present in the thalamus [54], we hypothesized that they would likely be activated in response to high frequency stimulation (>100 Hz), although evidence from human studies is lacking.

### 4.4. Limitations

As previously stated, the patient data used in this study provides a unique opportunity to study the response of neocortical dysplastic tissue to CMS. We also lacked data for CMS applied at intermediary frequencies between 100–150 Hz. The repeatability of this response has not yet been confirmed in other patients for the frequencies studied here. The study by Velasco et al. (2006), showed the efficacy of 60–130 Hz CMS in 49 epileptic patients [5]. But their study employed implanted stimulators that delivered stimulation for long periods, 1–9 years, which would then also involve long-term plasticity mechanisms.

Neural mass modeling is a useful technique when studying neural population dynamics, as reported in [35,92–94]. In this study, the main limitation is related to the identification of key model parameters. Indeed, model parameters were tuned manually based on the visual comparison of simulated and recorded SEEG signals. No quantitative study was performed to determine the optimal parameter set. The integration of a parameter optimization algorithm to fit the model dynamics to the patient data is of interest for future work. This would also improve the applicability of such a thalamocortical model for simulating patient-specific responses.

It was seen in the simulated response to CMS that the time taken for the dynamic parameters to recover to their initial values resulted in a delay in the recovery of interictal activity. This may have also been due to the restricted number of neural masses considered in this study. Although the excitatory subpopulations receive external noise inputs, it could be of interest to expand the number of populations to be considered.

Astrocytic glutamate release has been reported during high frequency stimulation [17,95], and this may play a role in the lack of suppression observed at 150 Hz. We did not include this in our work due to the added complexity in modeling astrocytes and associated neurotransmitter reuptake mechanisms. Inclusion of such mechanisms as well may have improved the simulated response at 150 Hz CMS. We have also not modeled the activity of GABA transporters for 50–100 Hz CMS, as literature regarding this was considered insufficient.

A potential subsequent step would be to evaluate the proposed mechanisms within a biological context, for example through an animal slice study. Thus, we hope this study provides the basis to frame a prospective experimental study.

## 5. Conclusion

Direct electrical stimulation of the brain is a potential therapeutic technique for treating epilepsy. Given the critical role of thalamocortical interaction on neocortical epileptic activity, thalamic stimulation could be an efficient method to suppress neocortical interictal discharges if the mechanisms of interaction are unveiled. In this study we developed a thalamocortical neural mass model to simulate CMS induced frequency-dependent neocortical responses, as recorded from a patient with focal cortical dysplasia. Our results showed an interplay between stimulation frequency, synaptic plasticity and extra-synaptic inhibition, and suggested that CMS could suppress the interictal activity when these mechanisms are activated for an optimal stimulation frequency. We believe that our model will encourage experimental studies to test our hypothesis regarding the role of extrasynaptic tonic inhibition induced by stimulation, and to develop others in the search for novel therapeutic strategies for improving the care of patients with epilepsy.

## Supporting information

S1 TextAppendix A: Computational model.Table A. Model parameter set. Fig A. Block diagram of the thalamic compartment and neocortical compartment. Fig B. Simulated excitatory postsynaptic potential (EPSP) summation. Fig C. Centromedian Nucleus (CMN). **Appendix B: Threshold for self-inhibition.** Fig D. GABA accumulation threshold for 150 Hz centromedian nucleus stimulation (CMS). **Appendix C: Example of re-simulated signals.** Fig E. Simulated thalamic and neocortical stereoencephalographic signals. **Appendix D: Dynamic parameter evolutions.** Table B. Value of the dynamic parameters at t=10s. Fig F. Variation of dynamic parameters during centromedian nucleus stimulation (CMS). Fig G. Variation of dynamic parameters with stimulation frequency. **Appendix E: Short term depression at 150 Hz centromedian nucleus stimulation.** Fig H. Short-term depression of synaptic gain for fast-kinetics inhibitory subpopulation at 150 Hz centromedian nucleus stimulation. **Appendix F: Combinations of mechanisms.** Fig I. Simulated neocortical response to 100 Hz centromedian nucleus stimulation, in various combinations of mechanisms. Table C. Effect of mechanisms on suppression of interictal activity. **Appendix G: Bifurcation analysis.** Table D. Bifurcation points for neocortical PYR PSP ($y_{0}$) versus thalamic drive ($y_{6}$). Table E. Bifurcation points for neocortical PYR PSP ($y_{0}$) versus depression to thalamic drive to Pyr (k). **Appendix H: Patient recording.** Fig J. StereoElectroEncephaloGraphic (SEEG) recording: thalamic and neocortical signals. **Appendix I: 150 Hz centromedian nucleus stimulation in the absence of self-inhibition and GABA transporter activity.** Fig K. Simulated neocortical response to 150 Hz centromedian nucleus stimulation (CMS) in the absence of key neurophysiologically relevant mechanisms. **Appendix J: Period Doubling in bifurcation analysis of neocortical sub-system to thalamic drive.** Fig L. Bifurcation diagram for neocortical sub-system to thalamic drive: Period doubling. **Appendix K: Simulated thalamic SEEG.** Fig M. Simulated thalamic StereoElectroEncephaloGram (SEEG) during centromedian nucleus stimulation (CMS). **Appendix L: Noise analysis.** Fig N. Effect of variation in input noise. Fig O. Number of interictal spikes in displayed signals for Fig 4. **Appendix M: Thalamic postsynaptic potential without applied mechanisms.** Fig P. Simulated thalamic postsynaptic potentials (PSPs). **Appendix N: PYR-PV-positive interneurons network.** Fig Q. Signal similarity measure: $h_{XY}^{2}$. Fig R. Neocortical StereoElectroEncephaloGram (SEEG) signal.(DOCX)
