# Supplementary material for: Computational modeling of frequency-dependent neocortical response to thalamic neurostimulation in epilepsy
Source: PLoS Comput Biol. 2025 Apr 28;21(4):e1012943. doi: 10.1371/journal.pcbi.1012943 (PMC12143575; doi:10.1371/journal.pcbi.1012943)
Supplement: S1 Text — Table A. Model parameter set. Fig A. Block diagram of the thalamic compartment and neocortical compartment. Fig B. Simulated excitatory postsynaptic potential (EPSP) summation. Fig C. Centromedian Nucleus (CMN). Appendix B: Threshold for self-inhibition. Fig D. GABA accumulation threshold for 150 Hz centromedian nucleus stimulation (CMS). Appendix C: Example of re-simulated signals. Fig E. Simulated thalamic and neocortical stereoencephalographic signals. Appendix D: Dynamic parameter evolutions. Table B. Value of the dynamic parameters at t=10s. Fig F. Variation of dynamic parameters during centromedian nucleus stimulation (CMS). Fig G. Variation of dynamic parameters with stimulation frequency. Appendix E: Short term depression at 150 Hz centromedian nucleus stimulation. Fig H. Short-term depression of synaptic gain for fast-kinetics inhibitory subpopulation at 150 Hz centromedian nucleus stimulation. Appendix F: Combinations of mechanisms. Fig I. Simulated neocortical response to 100 Hz centromedian nucleus stimulation, in various combinations of mechanisms. Table C. Effect of mechanisms on suppression of interictal activity. Appendix G: Bifurcation analysis. Table D. Bifurcation points for neocortical PYR PSP (y0) versus thalamic drive (y6). Table E. Bifurcation points for neocortical PYR PSP (y0) versus depression to thalamic drive to Pyr (k). Appendix H: Patient recording. Fig J. StereoElectroEncephaloGraphic (SEEG) recording: thalamic and neocortical signals. Appendix I: 150 Hz centromedian nucleus stimulation in the absence of self-inhibition and GABA transporter activity. Fig K. Simulated neocortical response to 150 Hz centromedian nucleus stimulation (CMS) in the absence of key neurophysiologically relevant mechanisms. Appendix J: Period Doubling in bifurcation analysis of neocortical sub-system to thalamic drive. Fig L. Bifurcation diagram for neocortical sub-system to thalamic drive: Period doubling. Appendix K: Simulated thalamic SEEG. Fig M. Simulated [file pcbi.1012943.s001.docx]

**S1 Text**

**Appendix A: Computational model**

The parameter set employed for this study is given in Table A. The connectivity parameters are labelled as $C_{SP_{1}-SP_{2}}$, where $SP_{1}$ corresponds to the efferent sub-population and $SP_{2}$ is the afferent sub-population. There are two uncorrelated Gaussian noise inputs to the TC and Pyr sub-populations, with distributions $p_{TC}\left( t \right)\sim N(\mu_{TC},\sigma_{TC})$ and $p_{C}\left( t \right)\sim N(\mu_{C},\sigma_{C})$, respectively (as shown in Fig A). They represent the nonspecific inputs applied to TC and Pyr from other sources. The sum of the post-synaptic potentials (PSPs) arriving at the TC and Pyr were the outputs of the model of the thalamic and neocortical compartments, respectively. The neocortical time constants were tuned based on [1, 2].

Regarding the model parameters, we would like to mention that we have focused on a reduced number of key parameters (n=14), namely those controlling the amplitude of excitatory PSPs (EPSPs) ($A_{C}, C_{B_{b}P}, C_{B_{a}P}, C_{GP}, \mu_{C}, \mu_{TC}, \sigma_{C}, \sigma_{TC}$) and inhibitory PSPs (IPSPs) ($B_{C}^{a}, B_{C}^{b}, G_{C}, C_{PG}, C_{PB}, C_{BG}$). The synaptic gains for the interneurons were reduced owing to the dysplastic nature of the recorded signal we were modeling. The time constants governing the rise and decay of PSPs were maintained within the physiologically relevant ranges, as in Fig B (although pyramidal time constants are higher than usual). The same strategy applies to the input/output function of each subpopulation, represented by a sigmoid function (gives the average firing rate of action potentials generated in a subpopulation as a function of its mean membrane potential). In summary, a set of 14 parameters (out of 85) have been tuned to minimise the difference between recorded and simulated StereoElectroEncephaloGraphy (SEEG) signals, based on visual inspection of the traces. The thalamo-cortical connections, and the thalamic time constants were based on the following literature: [3-10].

This neural mass modelling study was performed within the COALIA environment [11]. The stochastic differential equations (refer to S3) were iterated using the Euler-Maruyama method, with a time step of $dt=\frac{1}{10000} s$.

As described in section 2.3, based on the neural mass formalism, the h-function translates population spiking behavior to

**Table A. Model parameter set.**

|  | | Interpretation | Model parameter | Parameter value |
| --- | --- | --- | --- | --- |
| Neocortical synaptic gain parameters  (mV) | | Average excitatory neocortical synaptic gain | $\boldsymbol{A}_{\boldsymbol{C}}$ | 7.5 |
|  |  | Average synaptic gain for neocortical slow inhibitory basal synapses of SST interneuron (IN) | $\boldsymbol{B}_{\boldsymbol{C}}^{\boldsymbol{b}}$ | 20 |
|  |  | Average synaptic gain for neocortical slow inhibitory apical synapses of SST IN | $\boldsymbol{B}_{\boldsymbol{C}}^{\boldsymbol{a}}$ | 5 |
|  |  | Average synaptic gain of neocortical fast inhibitory PV IN | $\boldsymbol{G}_{\boldsymbol{C}}$ | 25 |
|  |  | Average inhibitory synaptic gain of neocortical VIP IN | $\boldsymbol{D}_{\boldsymbol{C}}$ | 5 |
|  |  | Average inhibitory synaptic gain of neocortical NGFC IN | $\boldsymbol{N}_{\boldsymbol{C}}$ | 10 |
|  |  |  |  |  |
| Thalamic synaptic gain parameters  (mV) | | Average synaptic gain of excitatory thalamic by TC | $\boldsymbol{A}_{\boldsymbol{Th}}$ | 3 |
|  |  | Average synaptic gain of thalamic slow inhibition by RtN2 | $\boldsymbol{B}_{\boldsymbol{Th}}$ | 10 |
|  |  | Average synaptic gain of thalamic fast inhibition by RtN1 | $\boldsymbol{G}_{\boldsymbol{Th}}$ | 20 |
|  |  | Average thalamic extrasynaptic tonic inhibitory gain | $\boldsymbol{E}_{\boldsymbol{T}}$ | 0.125 |
|  |  | Initial value for the average excitatory synaptic gain for depressed thalamo-pyramidal synapses | $\boldsymbol{A}_{\boldsymbol{C}}^{\boldsymbol{d}}$ | 3 |
| Neocortical synaptic time constants  $\boldsymbol{(}\boldsymbol{s}^{\boldsymbol{-1}}\boldsymbol{)}$ | | Average PYR EPSP rise time constant | $\boldsymbol{a}_{\boldsymbol{C}}^{\boldsymbol{1}}$ | 200 |
|  |  | Average PYR EPSP decay time constant | $\boldsymbol{a}_{\boldsymbol{C}}^{\boldsymbol{2}}$ | 180 |
|  |  | Average SST IN-basal connection’s IPSP rise time constant | $\boldsymbol{b}_{\boldsymbol{C}^{\boldsymbol{b}}}^{\boldsymbol{1}}$ | 100 |
|  |  | Average SST IN-basal connection’s IPSP decay time constant | $\boldsymbol{b}_{\boldsymbol{C}^{\boldsymbol{b}}}^{\boldsymbol{2}}$ | 100 |
|  |  | Average SST IN-apical IPSP rise time constant | $\boldsymbol{b}_{\boldsymbol{C}^{\boldsymbol{a}}}^{\boldsymbol{1}}$ | 21 |
|  |  | Average SST IN-apical IPSP decay time constant | $\boldsymbol{b}_{\boldsymbol{C}^{\boldsymbol{a}}}^{\boldsymbol{2}}$ | 21 |
|  |  | Average PV IN IPSP rise time constant | $\boldsymbol{g}_{\boldsymbol{C}}^{\boldsymbol{1}}$ | 600 |
|  |  | Average PV IN IPSP decay time constant | $\boldsymbol{g}_{\boldsymbol{C}}^{\boldsymbol{2}}$ | 400 |
|  |  | Average VIP IN IPSP rise time constant | $\boldsymbol{d}_{\boldsymbol{C}}^{\boldsymbol{1}}$ | 20 |
|  |  | Average VIP IN IPSP decay time constant | $\boldsymbol{d}_{\boldsymbol{C}}^{\boldsymbol{2}}$ | 20 |
|  |  | Average NGFC IN IPSP rise time constant | $\boldsymbol{n}_{\boldsymbol{C}}^{\boldsymbol{1}}$ | 5 |
|  |  | Average NGFC IN IPSP decay time constant | $\boldsymbol{n}_{\boldsymbol{C}}^{\boldsymbol{2}}$ | 5 |
| Thalamic synaptic time constants  $\boldsymbol{(}\boldsymbol{s}^{\boldsymbol{-1}}\boldsymbol{)}$ | | Average TC EPSP rise time constant | $\boldsymbol{a}_{\boldsymbol{Th}}^{\boldsymbol{1}}$ | 130 |
|  |  | Average TC EPSP decay time constant | $\boldsymbol{a}_{\boldsymbol{Th}}^{\boldsymbol{2}}$ | 70 |
|  |  | Average RtN2 IPSP rise time constant | $\boldsymbol{b}_{\boldsymbol{Th}}^{\boldsymbol{1}}$ | 400 |
|  |  | Average RtN2 IPSP decay time constant | $\boldsymbol{b}_{\boldsymbol{Th}}^{\boldsymbol{2}}$ | 20 |
|  |  | Average RtN1 IPSP rise time constant | $\boldsymbol{g}_{\boldsymbol{Th}}^{\boldsymbol{1}}$ | 1600 |
|  |  | Average RtN1 IPSP decay time constant | $\boldsymbol{g}_{\boldsymbol{Th}}^{\boldsymbol{2}}$ | 150 |
|  |  | Average extrasynaptic tonic inhibition rise time constant | $\boldsymbol{e}_{\boldsymbol{T}}^{\boldsymbol{1}}$ | 10 |
|  |  | Average extrasynaptic tonic inhibition decay time constant | $\boldsymbol{e}_{\boldsymbol{T}}^{\boldsymbol{2}}$ | 10 |
| Sigmoid function parameters | | Parameters of the nonlinear sigmoid function (transforming the average membrane potential into an average density of action potentials) | $\boldsymbol{\nu}_{\boldsymbol{0}}, \boldsymbol{e}_{\boldsymbol{0}},$  $\boldsymbol{r}$ | 6 mV, 2$\times$(2.5 $s^{-1}$),  0.56 m$V^{-1}$ |
| Connectivity constants | Pyr-Pyr | Average number of synaptic contacts between presynaptic and postsynaptic subpopulations | $\boldsymbol{C}_{\boldsymbol{P}_{\boldsymbol{1}}\boldsymbol{P}}$ | 110 |
|  | Pyr-Pyr |  | $\boldsymbol{C}_{\boldsymbol{P}\boldsymbol{P}_{\boldsymbol{1}}}$ | 135 |
|  | Pyr-PV |  | $\boldsymbol{C}_{\boldsymbol{PG}}$ | 30 |
|  | Pyr-SST |  | $\boldsymbol{C}_{\boldsymbol{PB}}$ | 60 |
|  | Pyr-TC |  | $\boldsymbol{C}_{\boldsymbol{PTC}}$ | 50 |
|  | PV-Pyr |  | $\boldsymbol{C}_{\boldsymbol{GP}}$ | 20 |
|  | PV-PV |  | $\boldsymbol{C}_{\boldsymbol{GG}}$ | 0 |
|  | SST-Pyr |  | $\boldsymbol{C}_{\boldsymbol{B}_{\boldsymbol{b}}\boldsymbol{P}}$ | 55 |
|  | SST-Pyr |  | $\boldsymbol{C}_{\boldsymbol{B}_{\boldsymbol{a}}\boldsymbol{P}}$ | 50 |
|  | SST-PV |  | $\boldsymbol{C}_{\boldsymbol{BG}}$ | 15 |
|  | NGFC-Pyr |  | $\boldsymbol{C}_{\boldsymbol{N}_{\boldsymbol{a}}\boldsymbol{P}}$ | 5 |
|  | VIP-SST |  | $\boldsymbol{C}_{\boldsymbol{DB}}$ | 5 |
|  | SST-VIP |  | $\boldsymbol{C}_{\boldsymbol{BD}}$ | 5 |
|  | Pyr-VIP |  | $\boldsymbol{C}_{\boldsymbol{PD}}$ | 5 |
|  | NGFC-NGFC |  | $\boldsymbol{C}_{\boldsymbol{NN}}$ | 6 |
|  | NGFC-VIP |  | $\boldsymbol{C}_{\boldsymbol{ND}}$ | 5 |
|  | TC-Pyr |  | $\boldsymbol{C}_{\boldsymbol{TCP}}$ | 32 |
|  | TC-TC |  | $\boldsymbol{C}_{\boldsymbol{TCTC}}$ | 40 |
|  | TC-PV |  | $\boldsymbol{C}_{\boldsymbol{TCG}}$ | 22 |
|  | TC-SST |  | $\boldsymbol{C}_{\boldsymbol{TCB}}$ | 15 |
|  | TC-NGFC |  | $\boldsymbol{C}_{\boldsymbol{TCN}}$ | 25 |
|  | TC-VIP |  | $\boldsymbol{C}_{\boldsymbol{TCD}}$ | 76 |
|  | TC-RtN1 |  | $\boldsymbol{C}_{\boldsymbol{TC}\boldsymbol{R}_{\boldsymbol{1}}}$ | 20 |
|  | TC-RtN2 |  | $\boldsymbol{C}_{\boldsymbol{TC}\boldsymbol{R}_{\boldsymbol{2}}}$ | 20 |
|  | Pyr- RtN1 |  | $\boldsymbol{C}_{\boldsymbol{P}\boldsymbol{R}_{\boldsymbol{1}}}$ | 10 |
|  | Pyr- RtN2 |  | $\boldsymbol{C}_{\boldsymbol{P}\boldsymbol{R}_{\boldsymbol{2}}}$ | 1 |
|  | RtN1-TC |  | $\boldsymbol{C}_{\boldsymbol{R}_{\boldsymbol{1}}\boldsymbol{TC}}$ | 15 |
|  | RtN2-TC |  | $\boldsymbol{C}_{\boldsymbol{R}_{\boldsymbol{2}}\boldsymbol{TC}}$ | 5 |
|  | |  | $\boldsymbol{C}_{\boldsymbol{ETC}}$ | 35 |
| Inter-compartmental propagation delay  (s) | |  | $\boldsymbol{d}_{\boldsymbol{P}-\boldsymbol{TC}}$ | 0.001 |
|  |  |  | $\boldsymbol{d}_{\boldsymbol{TC}-\boldsymbol{P}}$ | 0.002 |
| Neocortical noise parameters | |  | $\boldsymbol{\mu}_{\boldsymbol{C}}$ | 40 |
|  |  |  | $\boldsymbol{\sigma}_{\boldsymbol{C}}$ | 1.85 |
| Thalamic noise parameters | |  | $\boldsymbol{\mu}_{\boldsymbol{TC}}$ | 120 |
|  |  |  | $\boldsymbol{\sigma}_{\boldsymbol{TC}}$ | 3.5 |
| Coupling constants | TC | Electric field coupling constants | $\boldsymbol{k}_{\boldsymbol{Th}}^{\boldsymbol{TC}}$ | 4 |
|  | RtN1 |  | $\boldsymbol{k}_{\boldsymbol{Th}}^{\boldsymbol{R}\boldsymbol{1}}$ | 2 |
|  | RtN2 |  | $\boldsymbol{k}_{\boldsymbol{Th}}^{\boldsymbol{R}\boldsymbol{2}}$ | 2 |
| Parameters for short-term plasticity, Section 2.4 | STF at RtN2 to TC connection |  | $\boldsymbol{\tau}_{\boldsymbol{f}}^{\boldsymbol{R}\boldsymbol{2}}(s^{-1})$ | 10 |
|  |  |  | $\boldsymbol{\tau}_{\boldsymbol{d}}^{\boldsymbol{R}\boldsymbol{2}}(s^{-1})$ | 0.01 |
|  |  |  | $\boldsymbol{u}_{\boldsymbol{e}}^{\boldsymbol{R}\boldsymbol{2}}$ | 0.4 |
|  |  |  | $\boldsymbol{f}\boldsymbol{r}^{\boldsymbol{R}\boldsymbol{2}}$ | 2 |
|  | STD at RtN1 to TC connection |  | $\boldsymbol{\tau}_{\boldsymbol{f}}^{\boldsymbol{R}\boldsymbol{1}}(s^{-1})$ | 0.01 |
|  |  |  | $\boldsymbol{\tau}_{\boldsymbol{d}}^{\boldsymbol{R}\boldsymbol{1}}(s^{-1})$ | 0.8 |
|  |  |  | $\boldsymbol{u}_{\boldsymbol{e}}^{\boldsymbol{G}}$ | 0.05 |
|  |  |  | $\boldsymbol{f}\boldsymbol{r}^{\boldsymbol{R}\boldsymbol{1}}$ | 2 |
|  | STD at TC to Pyr connection |  | $\boldsymbol{\tau}_{\boldsymbol{f}}^{\boldsymbol{P}}(s^{-1})$ | 0.01 |
|  |  |  | $\boldsymbol{\tau}_{\boldsymbol{d}}^{\boldsymbol{P}}(s^{-1})$ | 10 |
|  |  |  | $\boldsymbol{u}_{\boldsymbol{e}}^{\boldsymbol{P}}$ | 0.2 |
|  |  |  | $\boldsymbol{f}\boldsymbol{r}^{\boldsymbol{P}}$ | 4.99 |
| Thalamic GABA reuptake time constant $\boldsymbol{(}\boldsymbol{s}^{\boldsymbol{-1}}\boldsymbol{)}$ | |  | $\boldsymbol{\tau}_{\boldsymbol{IE}}$ | 30 |


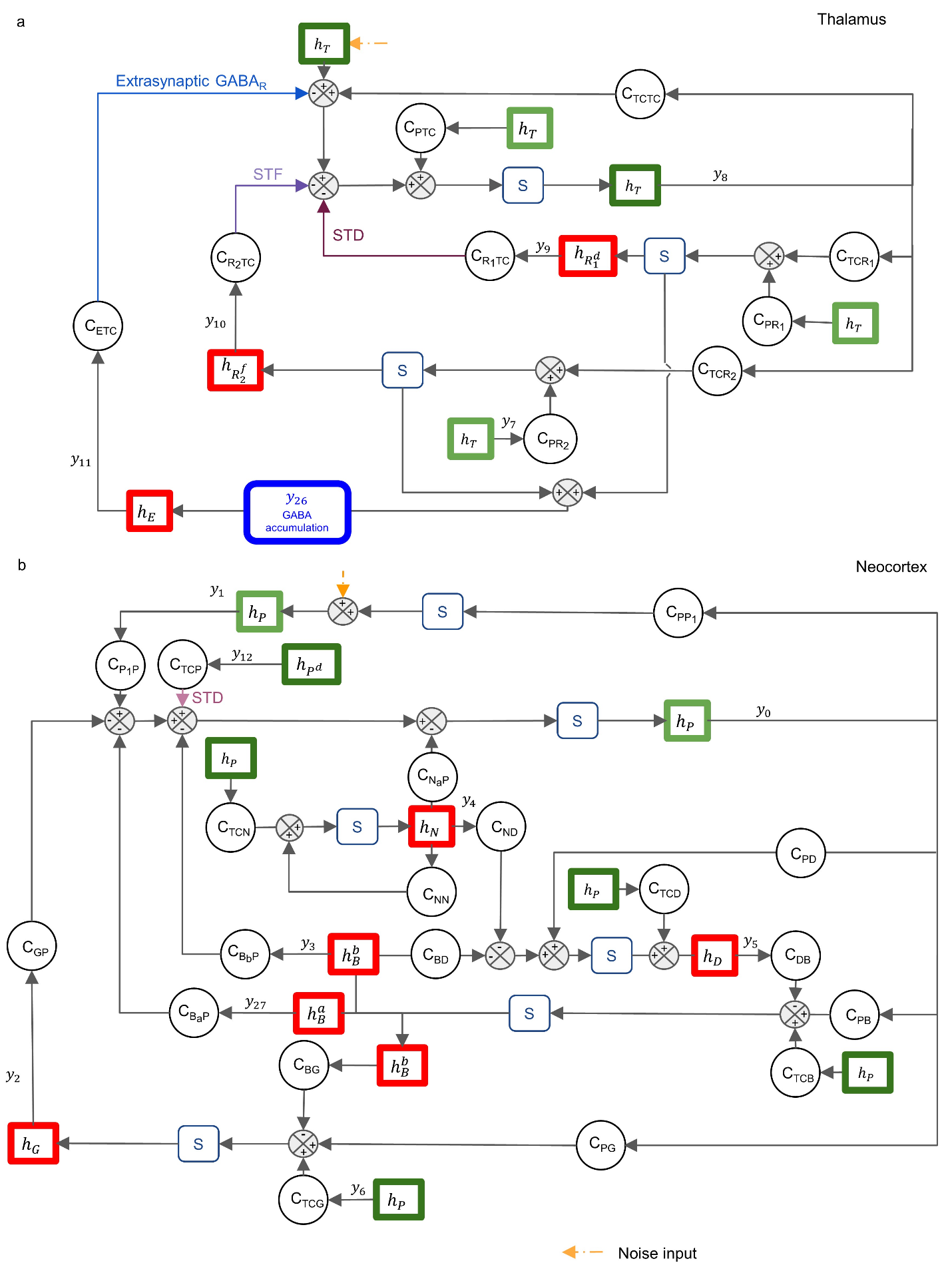


**Fig A. Block diagram of the thalamic compartment and neocortical compartment.**

(a) The thalamic compartment was composed of thalamic cells sub-population ($h_{T}$), inhibitory sub-population with fast dynamics ($h_{R_{1}^{d}}$) and inhibitory sub-population with slow dynamics ($h_{R_{2}^{f}}$). Neocortical inputs were applied to this compartment through the $h_{T}$. The “S” block denotes the wave-to-pulse sigmoid function. The connectivity constants are denoted as $C_{SP_{1}-SP_{2}}$, where $SP_{1}$ corresponds to the efferent sub-population and $SP_{2}$ is the afferent sub-population. The output from the h-function is denoted by $y_{i}$, where $"i"$ is the index. (b) The neocortical compartment was composed of pyramidal sub-population ($h_{P}$; STD thalamocortical $h_{P^{d}}$), inhibitory somatostatin-positive (apical: $h_{B}^{a}$, and basal: $h_{B}^{b}$), parvalbumin positive ($h_{G}$), vasoactive intestinal polypeptide positive ($h_{D}$) and neuroglia form cells ($h_{N}$) interneuron sub-populations. (STD: Short term depression, STF: Short term facilitation)


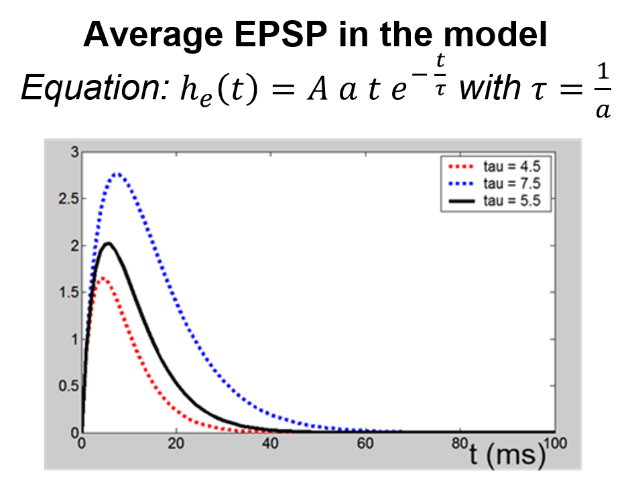


**Fig B. Simulated excitatory postsynaptic potential (EPSP) summation.**

Simulated average EPSPs with different time constant, tau (as shown), computed with the equation for the h-function (pulse-to-wave function).

The h-function for a given subpopulation is defined as $h=X e^{x_{2}\left( x_{1} \frac{\log\left( \frac{x_{2}}{x_{1}} \right)}{x_{2}-x_{1}}-1 \right)}$, where $X$ is the synaptic gain, and $(x_{1},x_{2})$ are the average synaptic time constants of the subpopulation coresponding to rise and decay times, respectively.

**First order differential equation**

The first order differential equations used in this neural mass model are as follows:

| PSP in PYR | $\dot{\boldsymbol{y}_{\boldsymbol{0}}}\boldsymbol{=}\boldsymbol{y}_{\boldsymbol{13}}$ $\dot{\boldsymbol{y}_{\boldsymbol{13}}}\boldsymbol{=}\boldsymbol{h}_{\boldsymbol{P}}\boldsymbol{S}\left( \boldsymbol{C}_{\boldsymbol{P}_{\boldsymbol{1}}\boldsymbol{P}}\boldsymbol{y}_{\boldsymbol{1}}\boldsymbol{+}\boldsymbol{C}_{\boldsymbol{TCP}} \boldsymbol{y}_{\boldsymbol{12}}\boldsymbol{-}\boldsymbol{C}_{\boldsymbol{GP}} \boldsymbol{y}_{\boldsymbol{2}}\boldsymbol{-}\boldsymbol{C}_{\boldsymbol{B}_{\boldsymbol{b}}\boldsymbol{P}} \boldsymbol{y}_{\boldsymbol{3}}\boldsymbol{-}\boldsymbol{C}_{\boldsymbol{B}_{\boldsymbol{a}}\boldsymbol{P}} \boldsymbol{y}_{\boldsymbol{27}}\boldsymbol{-}\boldsymbol{C}_{\boldsymbol{N}_{\boldsymbol{a}}\boldsymbol{P}} \boldsymbol{y}_{\boldsymbol{4}} \right)\boldsymbol{-}\left( \boldsymbol{a}_{\boldsymbol{C}}^{\boldsymbol{1}}\boldsymbol{+}\boldsymbol{a}_{\boldsymbol{C}}^{\boldsymbol{2}} \right)\boldsymbol{y}_{\boldsymbol{13}}\boldsymbol{-}\boldsymbol{a}_{\boldsymbol{C}}^{\boldsymbol{1}} \boldsymbol{a}_{\boldsymbol{C}}^{\boldsymbol{2}} \boldsymbol{y}_{\boldsymbol{0}}$ |
| --- | --- |
| PYR PSP due to PYR input | $\dot{y_{1}}=y_{14}$ $\dot{y_{14}}=h_{P} \left( P_{P}\left( t \right)+S\left( C_{PP_{1}} y_{0} \right) \right)-\left( a_{C}^{1}+a_{C}^{2} \right)y_{14}-a_{C}^{1} a_{C}^{2}y_{1}$ |
| PV PSP | $\dot{y_{2}}=y_{15}$ $\dot{y_{15}}=h_{G} S\left( C_{PG} y_{0}+C_{TCG} y_{6}-C_{BG} y_{3} \right)-\left( g_{C}^{1}+g_{C}^{2} \right)y_{15}-a_{C}^{1} a_{C}^{2}y_{2}$ |
| SST PSP (basal connection) | $\dot{y_{3}}=y_{16}$ $\dot{y_{16}}=h_{B}^{b} S\left( C_{PB} y_{0}+C_{TCB} y_{6}-C_{DB} y_{5} \right)-\left( b_{C^{b}}^{1}+b_{C^{b}}^{2} \right) y_{16}-b_{C^{b}}^{1} b_{C^{b}}^{2}y_{3}$ |
| NGFC PSP | $\dot{y_{4}}=y_{17}$ $\dot{y_{17}}=h_{N} S\left( C_{TCN} y_{6}-C_{NN} y_{4} \right)-\left( n_{C}^{1}+n_{C}^{2} \right) y_{17}-n_{C}^{1} n_{C}^{2}y_{4}$ |
| VIP PSP | $\dot{y_{5}}=y_{18}$ $\dot{y_{18}}=h_{D} S\left( C_{PD} y_{0}+C_{TCD} y_{6}-C_{BD} y_{3}-C_{ND} y_{4} \right)-\left( d_{C}^{1}+d_{C}^{2} \right)y_{18}-d_{C}^{1} d_{C}^{2}y_{5}$ |
| Thalamocortical PSP due to thalamic drive | $\dot{y_{6}}=y_{19}$ $\dot{y_{19}}=h_{P} S\left( {{A_{Th}a_{Th}P}_{Th}\left( t \right)+C}_{TCTC} y_{8}+C_{PTC} y_{7}-C_{R_{1}TC} y_{9}-C_{R_{2}TC} y_{10}-C_{ETC} y_{11} \right)-\left( a_{C}^{1}+a_{C}^{2} \right)y_{19}-a_{C}^{2}y_{6}$ |
| Corticothalamic PSP due to neocortical drive | $\dot{y_{7}}=y_{20}$ $\dot{y_{20}}=h_{T} S\left( C_{P_{1}P}y_{1}+C_{TCP} y_{12}-C_{GP} y_{2}-C_{B_{b}P} y_{3}-C_{B_{a}P} y_{27}-C_{N_{a}P} y_{4} \right)-\left( a_{Th}^{1}+a_{Th}^{2} \right)y_{20}-a_{Th}^{1} a_{Th}^{2}y_{7}$ |
| TC PSP | $\dot{y_{8}}=y_{21}$ $\dot{y_{21}}=h_{T} S\left( {{A_{Th}a_{Th}P}_{Th}\left( t \right)+C}_{TCTC} y_{8}+C_{PTC}y_{7}-C_{R_{1}TC} y_{9}-C_{R_{2}TC} y_{10}-C_{ETC} y_{11} \right)-\left( a_{Th}^{1}+a_{Th}^{2} \right)y_{21}-a_{Th}^{1} a_{Th}^{2}y_{8}$ |
| RtN2 PSP | $\dot{y_{9}}=y_{22}$ $\dot{y_{22}}=h_{R_{2}^{f}} S\left( C_{TCR_{1}} y_{8}+C_{PR_{1}} y_{7} \right)-\left( b_{Th}^{1}+b_{Th}^{2} \right)y_{22}-b_{Th}^{1} b_{Th}^{2}y_{9}$ |
| RtN1 PSP | $\dot{y_{10}}=y_{23}$ $\dot{y_{23}}= h_{R_{1}^{d}} S\left( C_{TCR_{2}} y_{8}+C_{PR_{2}}y_{7} \right)-\left( g_{Th}^{1}+g_{Th}^{2} \right)y_{23}-g_{Th}^{1} g_{Th}^{2}y_{10}$ |
| Extrasynaptic tonic inhibition potential | $\dot{y_{11}}=y_{24}$ $\dot{y_{24}}=h_{E} \left( y_{26} \right)-\left( e_{T}^{1}+e_{T}^{2} \right) y_{24}-e_{T}^{1} e_{T}^{2}y_{11}$ |
| Depressed thalamocortical PSP | $\dot{y_{12}}=y_{25}$ $\dot{y_{25}}=h_{P^{d}} S\left( {{A_{Th}a_{Th}P}_{Th}\left( t \right)+C}_{TCTC}y_{8}+C_{PTC} y_{7}-C_{R_{1}TC}y_{9}-C_{R_{2}TC} y_{10}-C_{ETC} y_{11} \right)-\left( a_{C}^{1}+a_{C}^{2} \right)y_{25}-a_{C}^{1} a_{C}^{2}y_{12}$ |
| SST PSP (apical connection) | $\dot{y_{27}}=y_{28}$ $\dot{y_{28}}= h_{B}^{a} S\left( C_{PB}y_{0}+C_{TCB}y_{6}-C_{DB}y_{5} \right)-\left( b_{C^{a}}^{1}+b_{C^{a}}^{2} \right)y_{28}-b_{C^{a}}^{1} b_{C^{a}}^{2} y_{27}$ |
| Where  $\boldsymbol{S(v)=}\frac{\boldsymbol{2}\boldsymbol{e}_{\boldsymbol{0}}}{\boldsymbol{[1+}\boldsymbol{e}^{\boldsymbol{r}\left( \boldsymbol{v}_{\boldsymbol{0}}\boldsymbol{-v} \right)}\boldsymbol{]}}$  $\boldsymbol{v}$ is the postsynaptic potential. This equation relates the average PSP of a sub-population to its average firing rate. | |


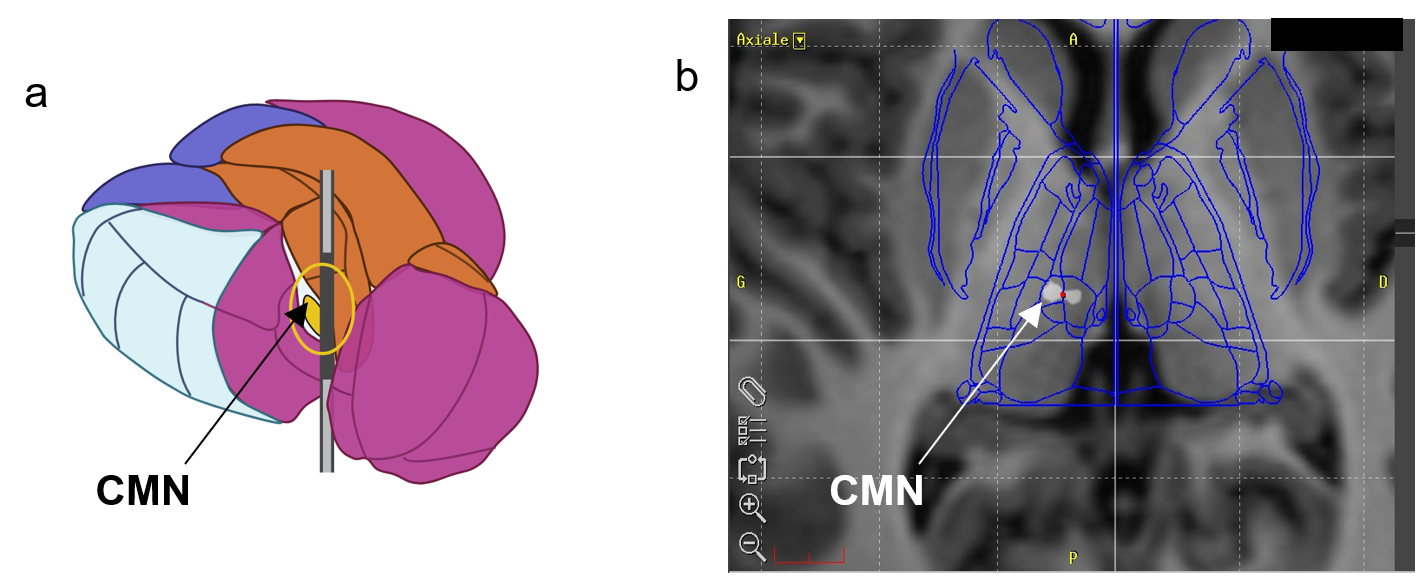


**Fig C. Centromedian Nucleus (CMN).**

(a) Illustration of the position of the CMN in the set of thalamic nuclei, with the StereoElectroEncephaloGram electrode contact positioned near it. (b) CMN (ovoid shape) and stimulation site (medial part of the CMN, red dot). With the Schaltenbrand and Wahren atlas map for CMN overlaid.

**Appendix B: Threshold for self-inhibition**

To simulate the activation of self-inhibition due to the activation of presynaptic GABAB receptors, we followed the GABA accumulation observed during 100 Hz CMS. From Fig D, it was seen that GABA accumulation was at 2.57 arbitrary units (A.U.). During 150 Hz CMS, GABA accumulation near the pre-synaptic membrane would occur rapidly owing to the higher firing rate of the subpopulation. Based on this, we increased $u_{e}$ at 8.35 s (to $u_{e}^{R1}$=2.5 and $u_{e}^{R2}$=20), when GABA accumulation surpassed 2.6 A.U. Thus, GABA accumulation was limited during 150 Hz CMS.


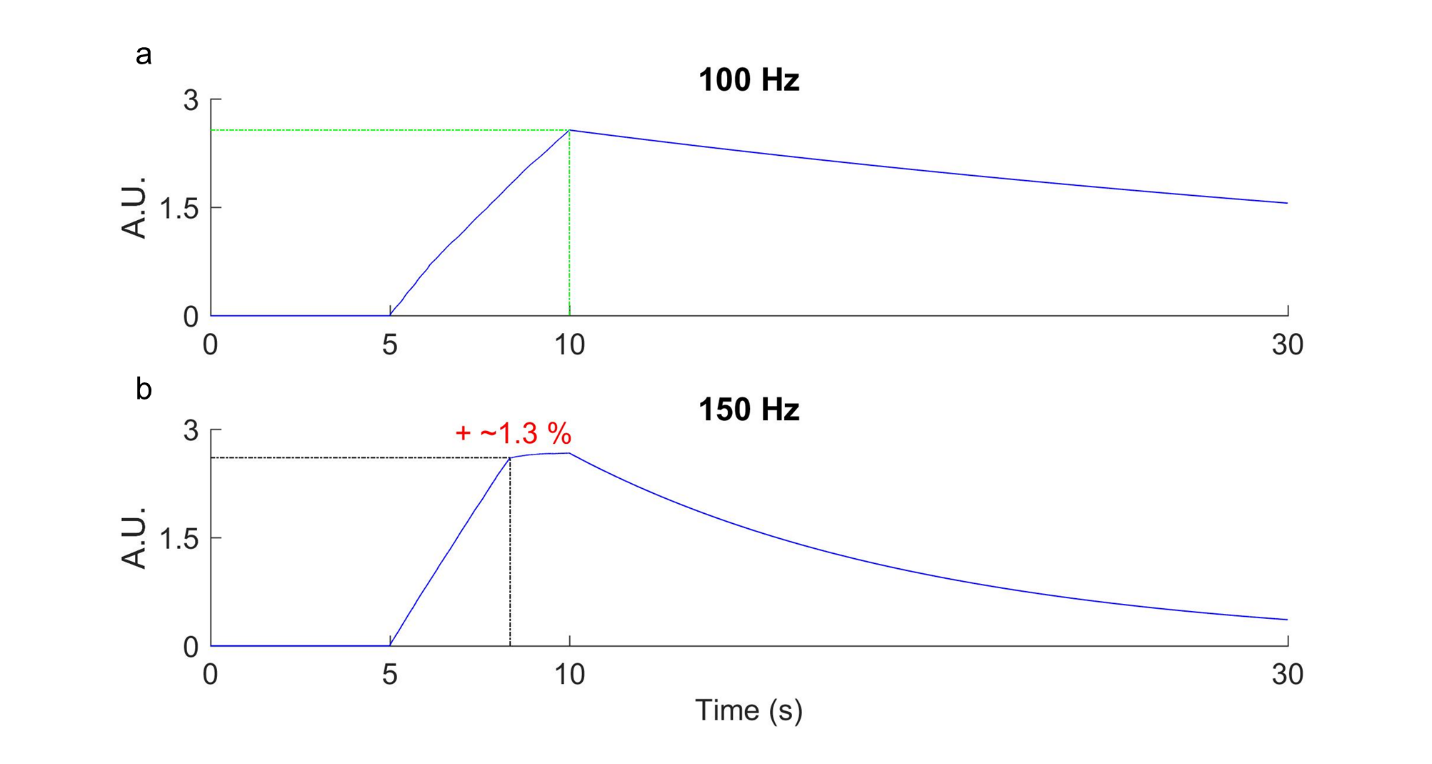


**Fig D. GABA accumulation threshold for 150 Hz centromedian nucleus stimulation (CMS).**

(a) During 100Hz CMS, GABA accumulation reaches a peak value of 2.57 arbitrary units (A.U., marked with green dotted lines) at the end of the stimulation period, from 5 to 10 seconds (s). (b) During 150 Hz CMS, when GABA accumulation surpasses ~1.3 % of the GABA accumulation value at 100 Hz CMS (8.35 s, marked with black dotted lines), self-inhibition was applied by increasing $u_{e}$ of the inhibitory sub-populations in the thalamic compartment.

**Appendix C: Example of re-simulated signals**

Here we plot simulated thalamic and neocortical signals to depict the effect of change in input noise to the thalamic and neocortical compartments. On visually comparing these signals to that presented in the results section of this study, differences in the period of suppression of interictal activity following stimulation could be noted.


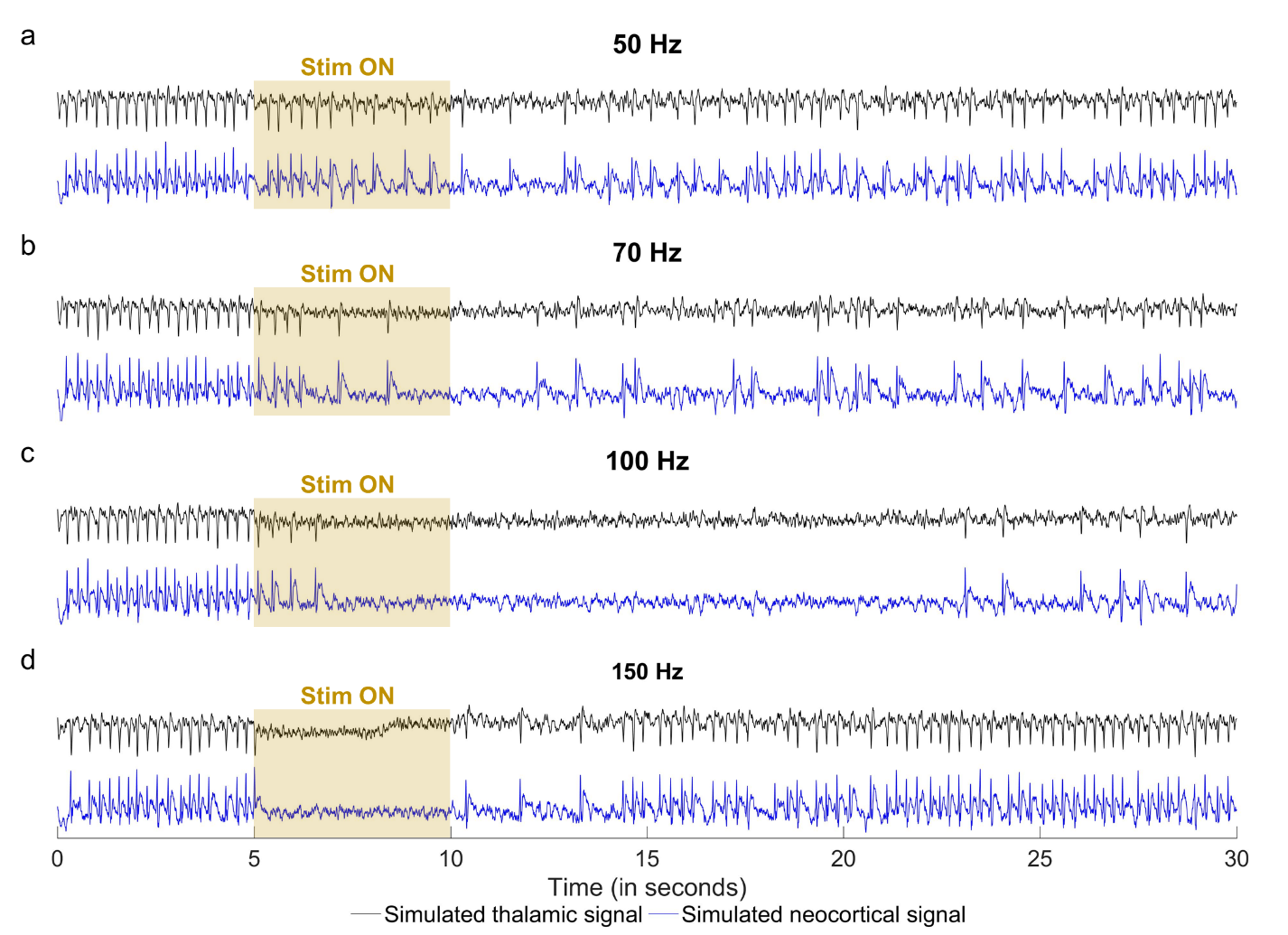


**Fig E. Simulated thalamic and neocortical stereoencephalographic signals.**

Thalamic stimulation was applied at (a) 50 Hz, (b) 70 Hz, (c) 100 Hz, and (d) 150 Hz during the Stim ON period, 5-10 s.

**Appendix D: Dynamic parameter evolutions**

One of the advantages of computational neural mass modelling is that we can track the dynamic parameters. The dynamic parameters implemented in our model varied with time in response to the stimulation applied. In this section, interested readers can closely observe how the dynamic parameters evolved in response to stimulation.

From Table B, it may be noted that synaptic gain of RtN1 did not vary significantly for stimulation in the range 50-100 Hz. But at 150 Hz CMS, $G_{d}^{Th}$ became 0.6597 normalized units at $t=10s$ following stimulation. This can be attributed to the parameter changes implemented at this stimulation frequency (Section 2.6).

**Table B. Value of the dynamic parameters at** $\boldsymbol{t=10}\boldsymbol{s}$**.**

| Stimulation Frequency  (Hz) | Parameter value at $t=10s$ | | | | | | | |
| --- | --- | --- | --- | --- | --- | --- | --- | --- |
|  | $G_{d}^{Th}$ | | $B_{f}^{Th}$ | | $Th_{GABA}$ | | $A_{d}^{C}$ | |
|  | N | MP | N | MP | N | MP | N | MP |
| 50 | 0.9921 | 19.93 | 0.8871 | 12.16 | 0.5333 | 1.42 | 0.2601 | 7.10 |
| 70 | 0.9913 | 19.91 | 0.9245 | 12.66 | 0.7021 | 1.87 | 0.2553 | 7.09 |
| 100 | 0.9906 | 19.90 | 0.9753 | 13.34 | 0.9636 | 2.57 | 0.2414 | 7.08 |
| 150 | 0.6597 | 13.45 | 0.0302 | 0.67 | 0.9998 | 2.66 | 0.0142 | 6.95 |

N: Normalized parameter value; MP: Model parameter value, in arbitrary units


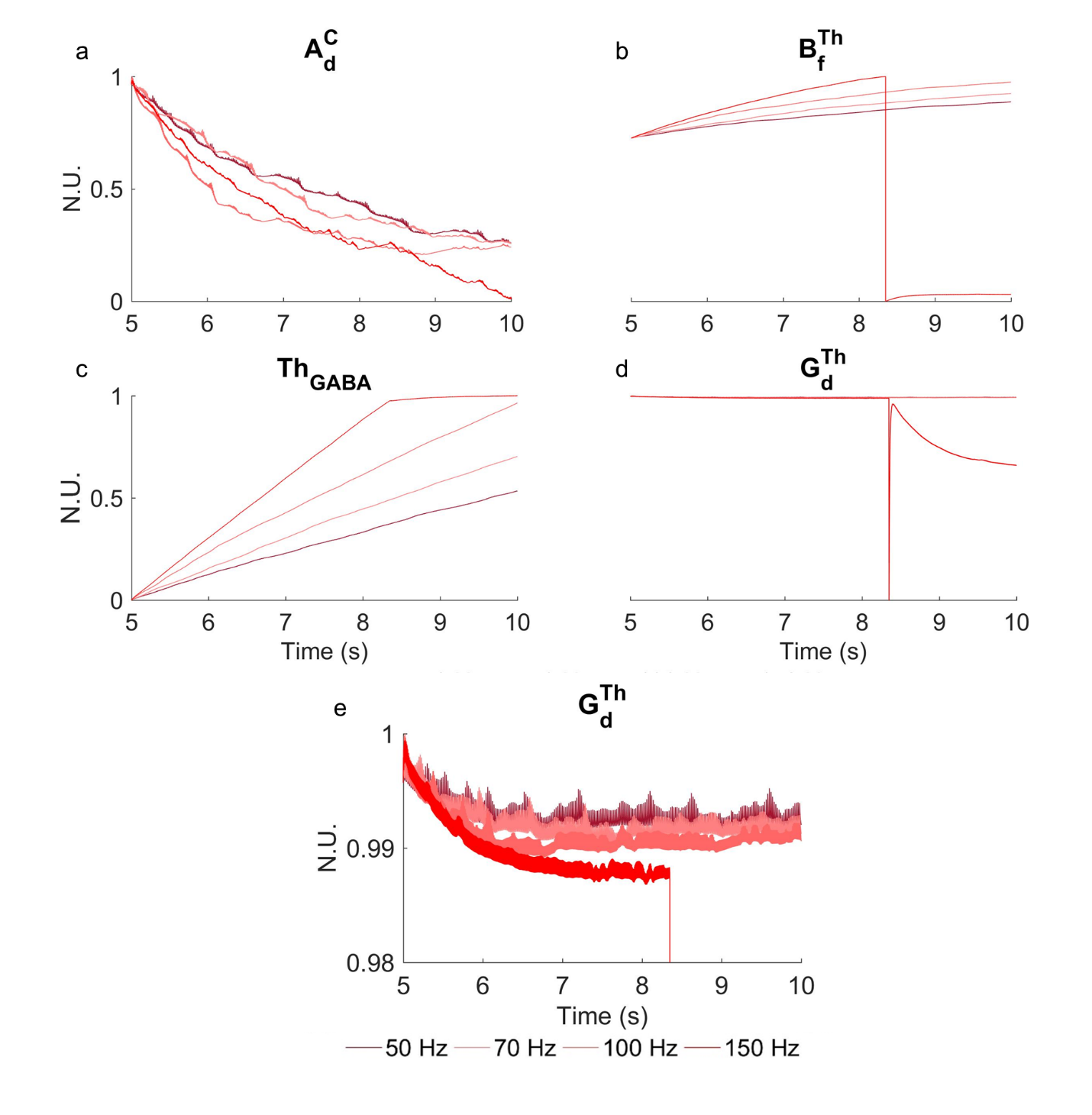


**Fig F. Variation of dynamic parameters during centromedian nucleus stimulation (CMS).**

(a) Synaptic gain of neocortical pyramidal subpopulation, $A_{d}^{C}$, decreased during CMS due to short-term depression (STD). The effect of STD was directly proportional to the stimulation frequency. (b) Synaptic gain of the thalamic slow dynamics subpopulation (RtN2), $B_{f}^{\mathrm{Th}}$, increased during CMS due to short-term facilitation (STF). The effect of STF was directly proportional to the stimulation frequency. During 150 Hz CMS, $u_{e}^{R2}$ was increased at 8.35 seconds (s) to simulate self-inhibition of the thalamic inhibitory subpopulations at this frequency. (c) GABA accumulation, $Th^{\mathrm{GABA}}$, increased during CMS. The accumulation was directly proportional to the stimulation frequency. At 150 Hz CMS, GABA accumulation saturated due to the activation of self-inhibition mechanisms. (d) Synaptic gain of the thalamic fast dynamics subpopulation (RtN1), $G_{d}^{\mathrm{Th}}$. During 150 Hz CMS, $u_{e}^{R1}$ subpopulation was increased at 8.35 s to simulate self-inhibition of the thalamic inhibitory subpopulations at this frequency, which resulted in its unique dynamics. (e) $G_{d}^{\mathrm{Th}}$, decreased during CMS due to STD. The effect of STD was directly proportional to the stimulation frequency, as can be seen in the zoomed in plot of (d). (N.U.: normalized units)


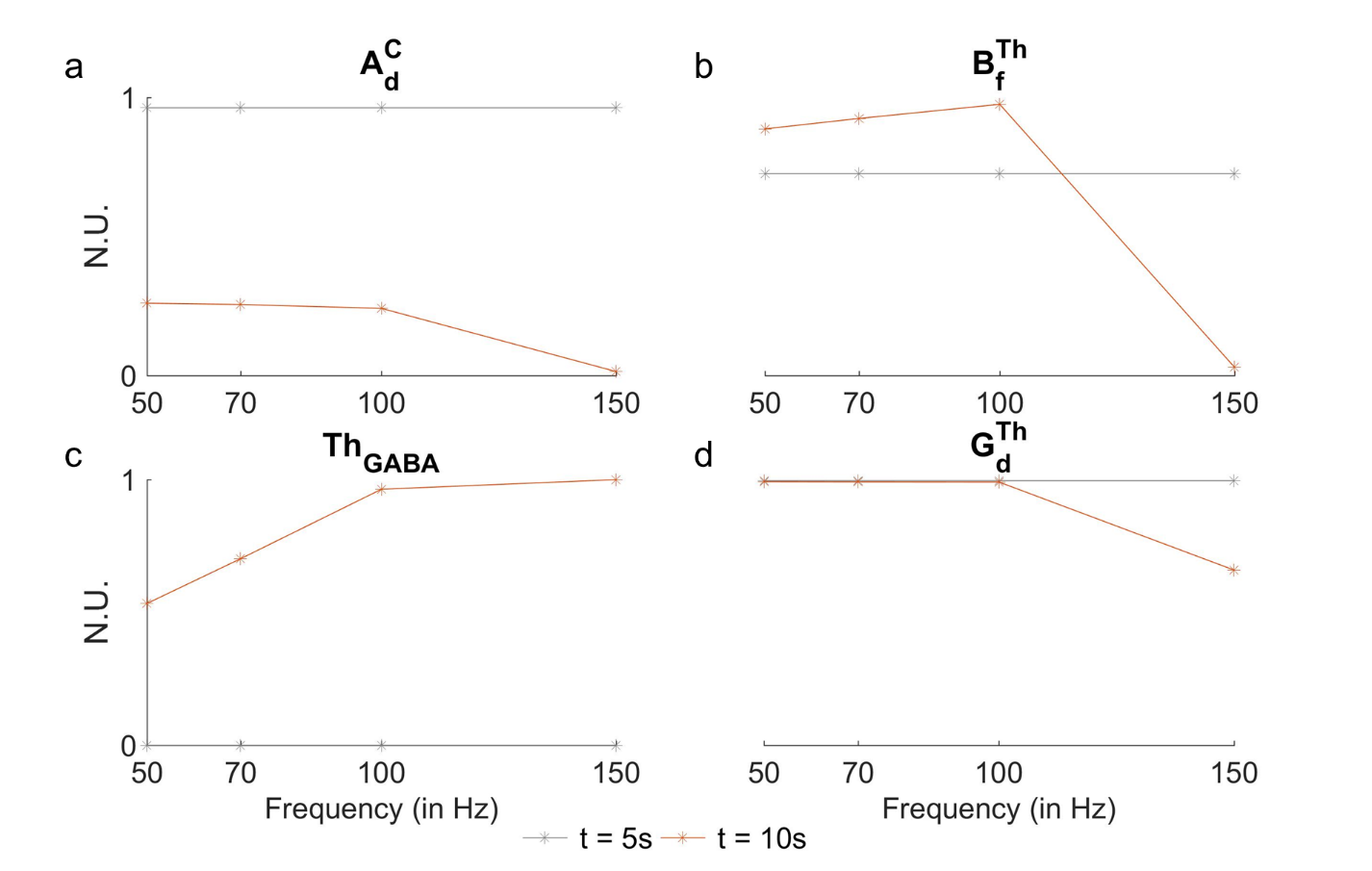


**Fig G. Variation of dynamic parameters with stimulation frequency.**

(a) The synaptic gain of neocortical pyramidal subpopulation, $A_{d}^{C}$, decreased from its value at 5s (in grey) to its value at 10s (in red) with higher stimulation frequency. (b) The synaptic gain of thalamic slow dynamics subpopulation (RtN2), $B_{f}^{\mathrm{Th}}$, increased from its value at 5s (in grey) to its value at 10s (in red) with higher stimulation frequency. (c) GABA accumulation, $Th^{\mathrm{GABA}}$, increased from its value at 5s (in grey) to its value at 10s (in red) with higher stimulation frequency. (d) The synaptic gain of thalamic fast dynamics subpopulation (RtN1), $G_{d}^{\mathrm{Th}}$, decreased from its value at 5s (in grey) to its value at 10s (in red) with higher stimulation frequency.

**Appendix E: Short term depression at 150 Hz centromedian nucleus stimulation.**

In the simulated response to 150 Hz CMS, the synaptic gain at the connection between the fast kinetics inhibitory subpopulation (RtN1) and the excitatory thalamic cells subpopulation (TC) in the thalamus ($G_{\mathrm{Th}}$) was subject to short-term depression, when the firing rate increased above the normal threshold. This was modelled as described in Section 2.4. But at 150 Hz CMS, we assumed that the rapid accumulation of GABA neurotransmitters near the presynaptic membrane would result in the activation of self-inhibition mechanisms in RtN1. This was modelled by a change in presynaptic release probability in the RtN1, $u_{e}^{R1}$, from 0.05 to 2.5 (in arbitrary units). But this resulted in an abrupt fall in $G_{\mathrm{Th}}$to 0.5912, after which it rapidly recovers to 19.23.

To understand the logic behind this change we must step through the short-term plasticity equations (STD) (1)-(3) presented in Section 2.4, and follow the evolution of the parameters involved, as shown in Fig H. When $u_{e}^{R1}$ was increased to 2.5, the first term in the right hand-side of $(1)$ increases, as $u^{R1}$ = 0.146. Also, as $\tau_{f}^{R1}=$0.01, the computation resulted in a rapid rise in $\frac{du}{dt}$ at this time step leading to the abrupt change in $G_{\mathrm{Th}}$. But in the following iteration $u^{R1}$ becomes ~2.47 (at 8.4 s). Thus, $G_{\mathrm{Th}}$becomes 19.23, with the continued action of STD during CMS.

Similarly, a shift in $B_{\mathrm{Th}}$ was also observed corresponding to the change in its $u_{e}^{R2}$ value. But as $\tau_{f}^{R2}$ was much greater than $\tau_{f}^{R1}$, it did not result in an abrupt/drastic change in $B_{\mathrm{Th}}$.


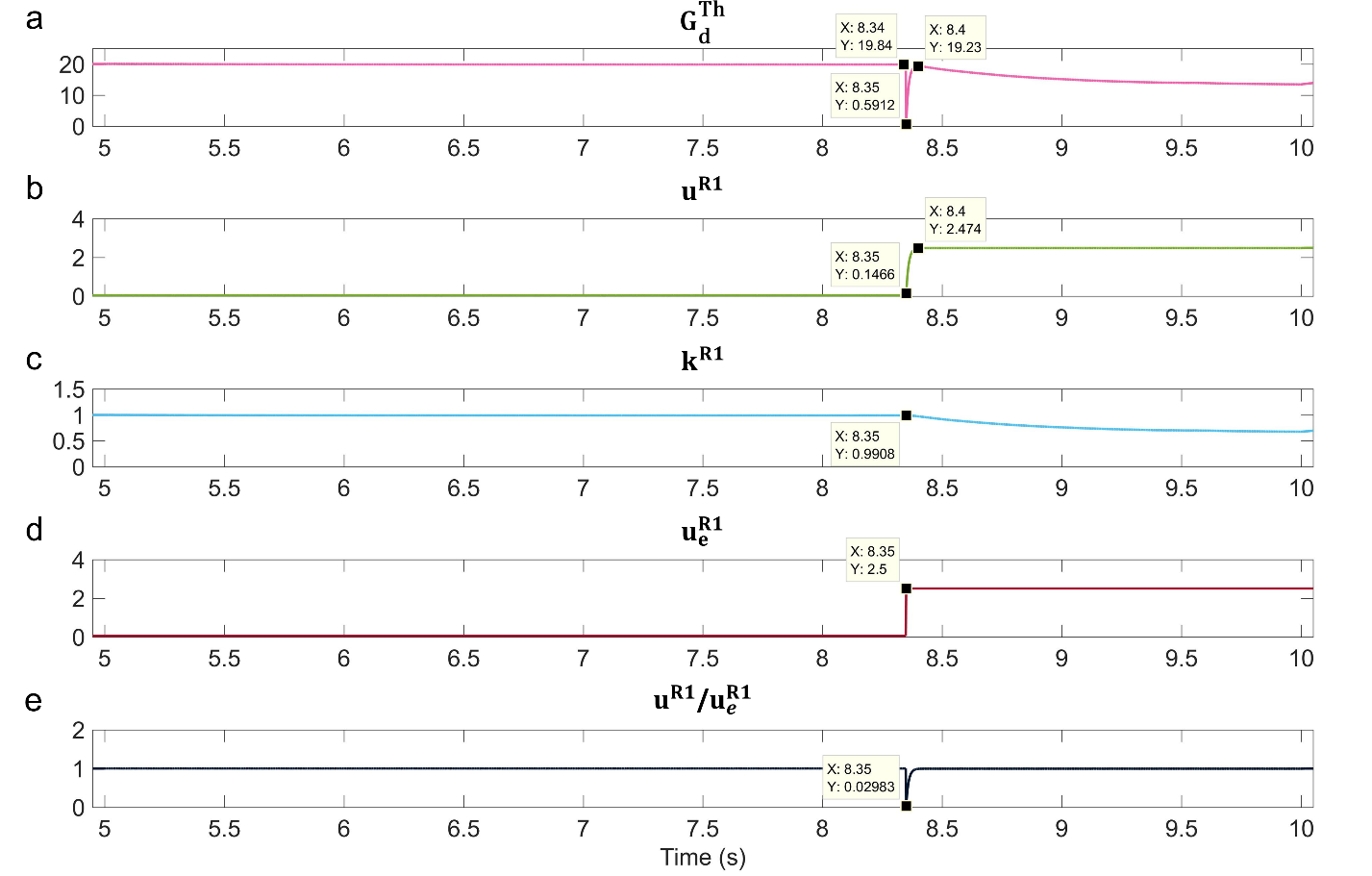


**Fig H. Short-term depression of synaptic gain for fast-kinetics inhibitory subpopulation at 150 Hz centromedian nucleus stimulation.**

(a) Plot of the evolution of the dynamic synaptic gain ($G_{\mathrm{Th}}$) corresponding to this subpopulation, with markers at 8.34, 8.35, and 8.4 s. (b) Plot of the fraction of resources utilized by each spike during synaptic depression, $u^{R1}$, with makers at 8.35 and 8.4s.(c) Plot of the amount of available resources, $k^{R1}$, with a marker at 8.35s. (d) Plot of the baseline fraction of resources utilized by each spike, $u_{e}^{R1}$, with marker at 8.35 s. (e) Plot of ${u^{R1}}/{u_{e}^{R1}}$, with marker at 8.35 s.

**Appendix F: Combinations of mechanisms**


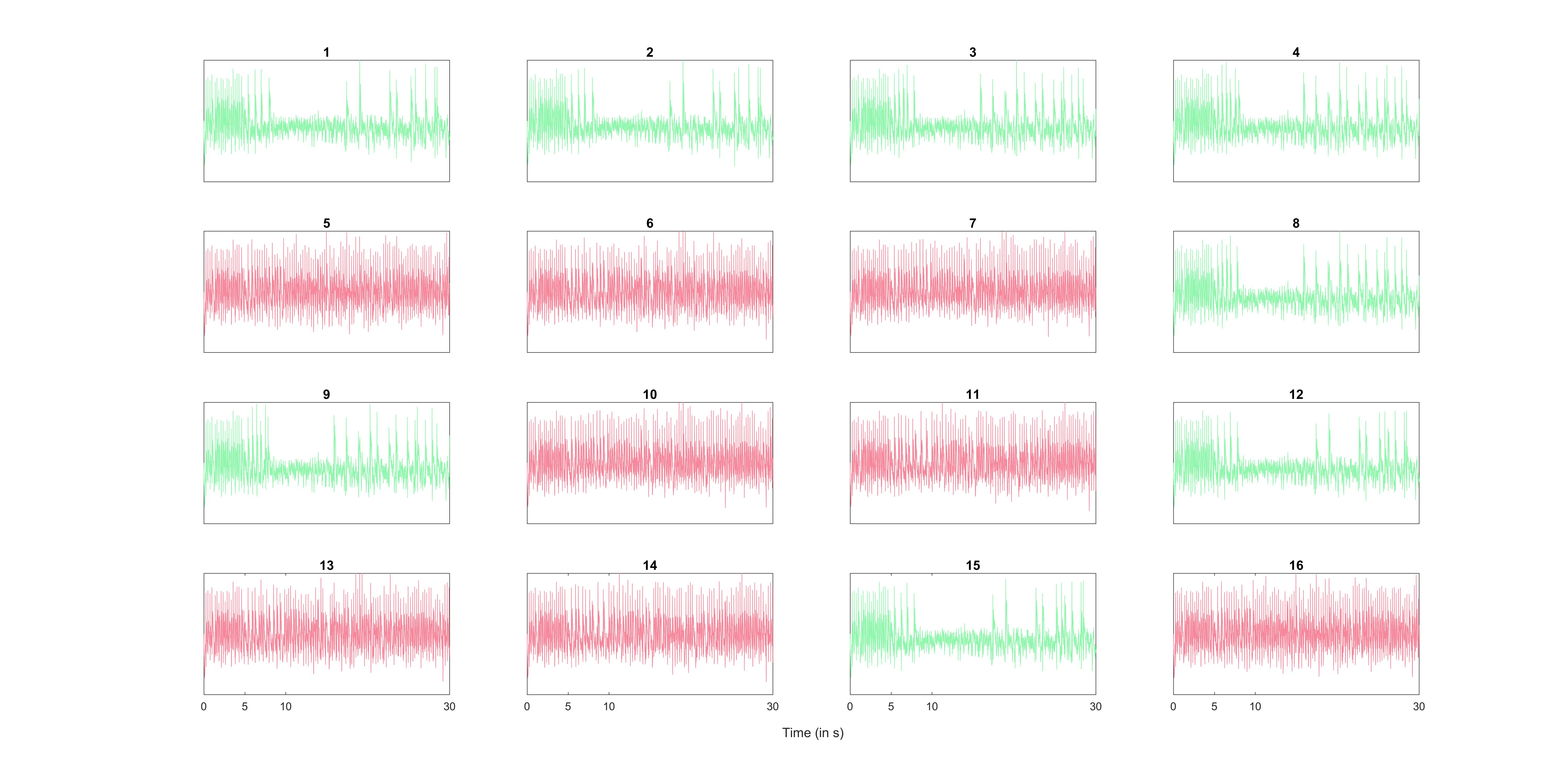


**Fig I. Simulated neocortical response to 100 Hz centromedian nucleus stimulation, in various combinations of mechanisms.**

The simulated neocortical responses, labelled 1-16. The plots in green: suppression of interictal activity following stimulation, and plots in red: continued interictal activity following stimulation. Stimulation was applied between 5-10 s (shaded region).

We ran different simulation studies to evaluate the influence of each of the various neurophysiological mechanisms we implemented in our study, in response to 100 Hz CMS. These mechanistic combinations are presented in Table C, and the simulated neocortical responses corresponding to each mechanistic scenario is presented in Fig I.

From Fig I, it can be seen that the simulation responses labelled 1, 2, 3, 4, 8, 9, 12 and 15 had a period of suppression of interictal activity following CMS, while the remaining simulation responses lacked this behavior. Hence, we classified the responses with suppression and without it into Class I and Class II, respectively. In Table C, for a given simulation labelled $i$, the mechanisms implemented correspond to the highlighted cells. For example, for the simulation 15, short-term facilitation of the slow-kinetics expressing inhibitory synapses onto thalamic cells subpopulation and extrasynaptic tonic inhibition were active.

**Table C. Effect of mechanisms on suppression of interictal activity.**

|  |  | **Mechanisms implemented** | | | |
| --- | --- | --- | --- | --- | --- |
|  |  | STD (RtN1) | STF (RtN2) | STD (TC to Pyr) | ExtraSynI |
| **Simulation label** | *1* |  |  |  |  |
|  | *2* |  |  |  |  |
|  | *3* |  |  |  |  |
|  | *4* |  |  |  |  |
|  | *8* |  |  |  |  |
|  | *12* |  |  |  |  |
|  | *15* |  |  |  |  |
|  | *9* |  |  |  |  |
|  | *5* |  |  |  |  |
|  | *6* |  |  |  |  |
|  | *7* |  |  |  |  |
|  | *10* |  |  |  |  |
|  | *11* |  |  |  |  |
|  | *13* |  |  |  |  |
|  | *14* |  |  |  |  |
|  | *16* |  |  |  |  |

| Class I: | Suppression of interictal spiking during/after stimulation |
| --- | --- |
| Class II: | Interictal spiking during stimulation |

| STD (RtN1): | Short-term depression of fast-kinetics inhibitory synapses onto thalamic cells sub-population |
| --- | --- |
| STF (RtN2): | Short-term facilitation of slow-kinetics inhibitory synapses onto thalamic cells sub-population |
| STD (TC to Pyr): | Short-term depression of thalamic cells’ synapses onto pyramidal cells sub-population |
| ExtraSynI: | Extrasynaptic tonic inhibition |

Among the combinations of mechanisms explored, we observed four significant simulation results. These were the simulations with: (1) extrasynaptic tonic inhibition (ExtraSynI), STF between RtN2-TC subpopulations, and STD between TC-PYR subpopulations; (2) ExtraSynI only; (3) ExtraSynI and STD between TC-Pyr subpopulations; and (4) ExtraSynI and STF between RtN2-TC subpopulations.

We qualitatively compared the simulated neocortical response from our model (Fig I) to that observed in each of these simulated responses. The similarity between the simulated neocortical response from simulation study (2) (with that of our model) indicated that the STD at the connection between RtN1 and TC had no significant impact on the model output. But when all the STP mechanisms were excluded, as in simulation (4), the suppression period was only 5.9 s. This showed that CMS-induced suppression of neocortical interictal discharges was not solely due to extra-synaptic tonic inhibition.

In simulation (8), where STF at the connection between RtN2 and TC was inactive, CMS induced suppression occurred during 8.1-15.5 s. While in simulation (12), where STD at the connection between TC and Pyr was inactive, CMS induced suppression was during 8.3-17.4s. This difference in suppression periods revealed that STF in the local inhibitory connection (STF(RtN2-TC)) had a stronger influence on the duration of the suppression period. It also showed that STD at the connection between TC and Pyr (STD(TC-Pyr)) influenced the onset of suppression of interictal activity.

**Appendix G: Bifurcation analysis**

Here we present the coordinates of the bifurcation points quoted in Section 3.4.

**Table D. Bifurcation points for neocortical PYR PSP (**$\boldsymbol{y}_{\boldsymbol{0}}$**) versus thalamic drive (**$\boldsymbol{y}_{\boldsymbol{6}}$**).**

| **Label** | $\boldsymbol{y}_{\boldsymbol{6}}$ | $\boldsymbol{y}_{\boldsymbol{0}}$ |
| --- | --- | --- |
| $HB_{1}^{y_{6}}$ | 0.1067 | 0.0183 |
| $HC_{1}^{y_{6}}$ | 0.1062 | 0.0267 |
| $HC_{2}^{y_{6}}$ | 0.1063 | 0.0265 |
| $SN_{1}^{y_{6}}$ | 0.1075 | 0.0217 |
| $SN_{2}^{y_{6}}$ | 0.0973 | 0.0473 |
| $HB_{2}^{y_{6}}$ | 0.1657 | 0.0987 |
| $HB_{3}^{y_{6}}$ | 0.2677 | 0.0865 |
| $SN_{3}^{y_{6}}$ | 0.3700 | 0.0459 |
| $SN_{4}^{y_{6}}$ | 0.3606 | 0.0278 |
| $HB_{4}^{y_{6}}$ | 0.3718 | 0.0192 |

**Table E. Bifurcation points for neocortical PYR PSP (**$\boldsymbol{y}_{\boldsymbol{0}}$**) versus depression to thalamic drive to Pyr (**$\boldsymbol{k}$**).**

| **Label** | $\boldsymbol{k}$ | $\boldsymbol{y}_{\boldsymbol{0}}$ |
| --- | --- | --- |
| $HB_{1}^{k}$ | 0.7352 | 0.0186 |
| $SN_{1}^{k}$ | 0.7365 | 0.0198 |
| $HC_{1}^{k}$ | 0.7307 | 0.0240 |
| $SN_{2}^{k}$ | 0.1695 | 0.0599 |
| $HB_{2}^{k}$ | 0.6564 | 0.0814 |
| $HB_{3}^{k}$ | 1.0904 | 0.0898 |
| $HB_{4}^{k}$ | 8.8384 | 0.1646 |

**Appendix H: Patient recording**

Here we present 30 s of StereoElectroEncephaloGraphic (SEEG) recording from the patient data to illustrate the variability in this subjects SEEG recording. As can be seen, the morphology and the frequency were inconsistent at various time-points, in this recording window. But the neocortical spike-wave complex appears to be the predominant feature in this patient’s recording.


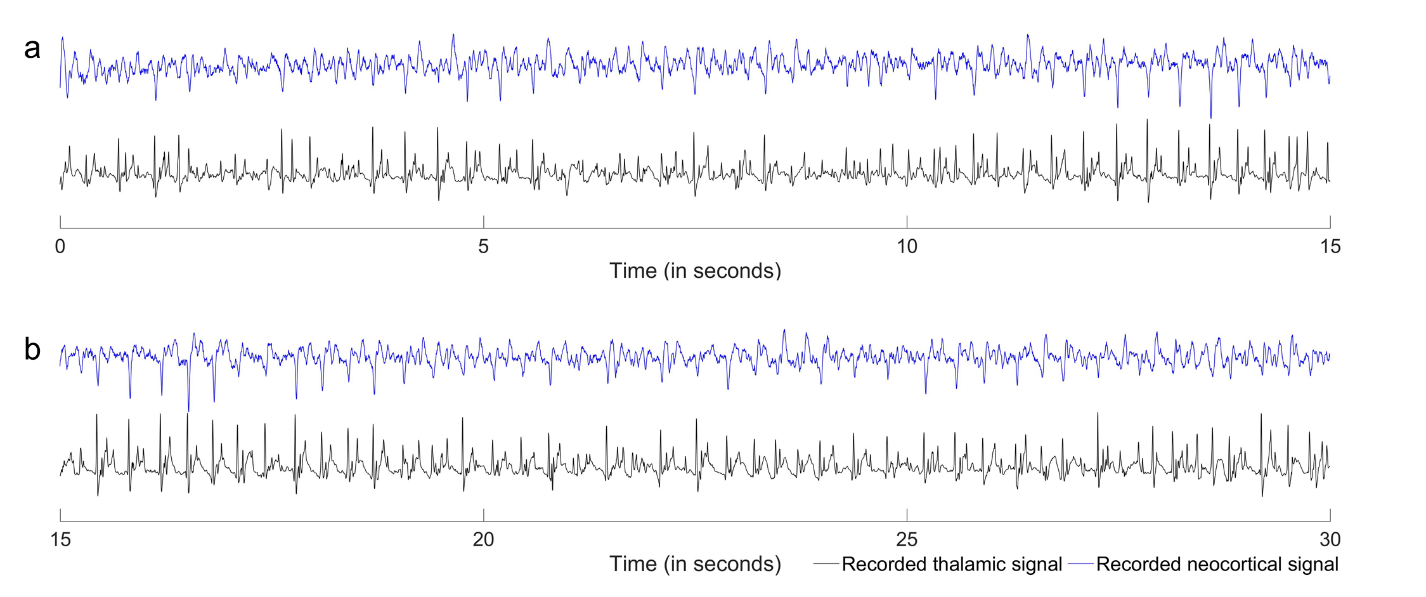


**Fig J. StereoElectroEncephaloGraphic (SEEG) recording: thalamic and neocortical signals.**

The SEEG recording from the thalamic and neocortical contacts in the patient (a) for a 15-seconds period, and (b) for the following 15-seconds period.

**Appendix I: 150 Hz centromedian nucleus stimulation in the absence of self-inhibition and GABA transporter activity**

In our study, to simulate neocortical response to centromedian stimulation at 150 Hz we have implemented certain neurophysiological mechanisms, as described in Section 2.6. Here we present the neocortical response in the absence of these mechanisms to illustrate its impact.

As shown in Fig K, in the absence of these mechanisms the effect of extrasynaptic tonic inhibition could last until ~24s post stimulation, after which interictal-spiking activity begins to reappear.


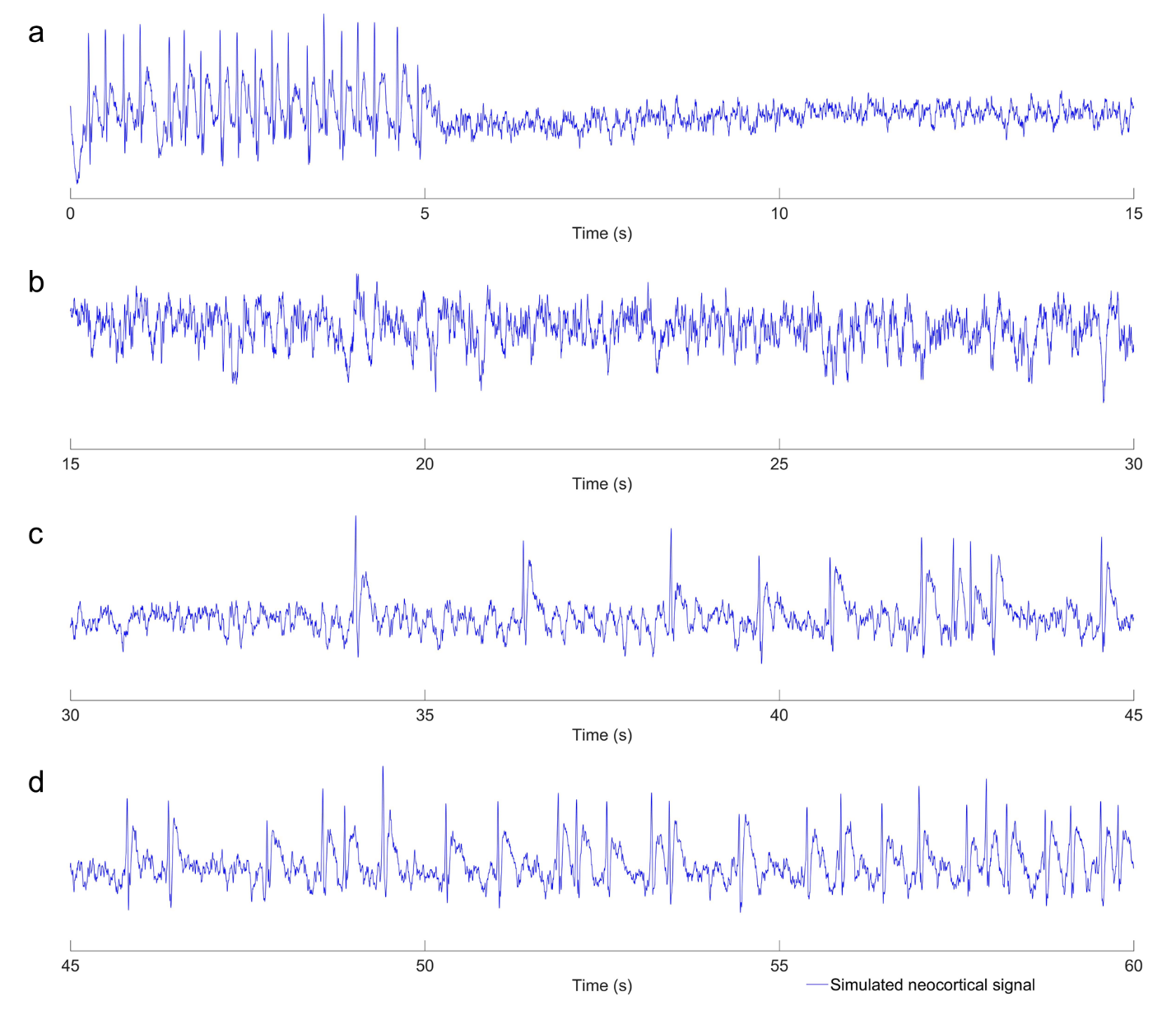


**Fig K. Simulated neocortical response to 150 Hz centromedian nucleus stimulation (CMS) in the absence of key neurophysiologically relevant mechanisms.**

The simulated neocortical response to stimulation in four-windows: (a) 0-15 s, (b) 15-30 s, (c) 30-45 s, and (d) 45-60 s, with stimulation at 5-10 s.

**Appendix J: Period Doubling in bifurcation analysis of neocortical sub-system to thalamic drive**

In the bifurcation analysis for the neocortical sub-system to thalamic drive (Fig La), a period doubling point was observed on the fixed solutions branch from $HB_{4}^{y_{6}}$, namely $PD_{1}^{y_{6}}$. Here, we illustrate the orbits for $y_{6}\approx0.37675$1. As shown in Fig Lb, two solutions exist for this, namely $y_{0}^{f}$ and $y_{0}^{PD}$, on the limit cycles branch and the period doubling branch, respectively. The corresponding orbits were as shown in Fig Lc.


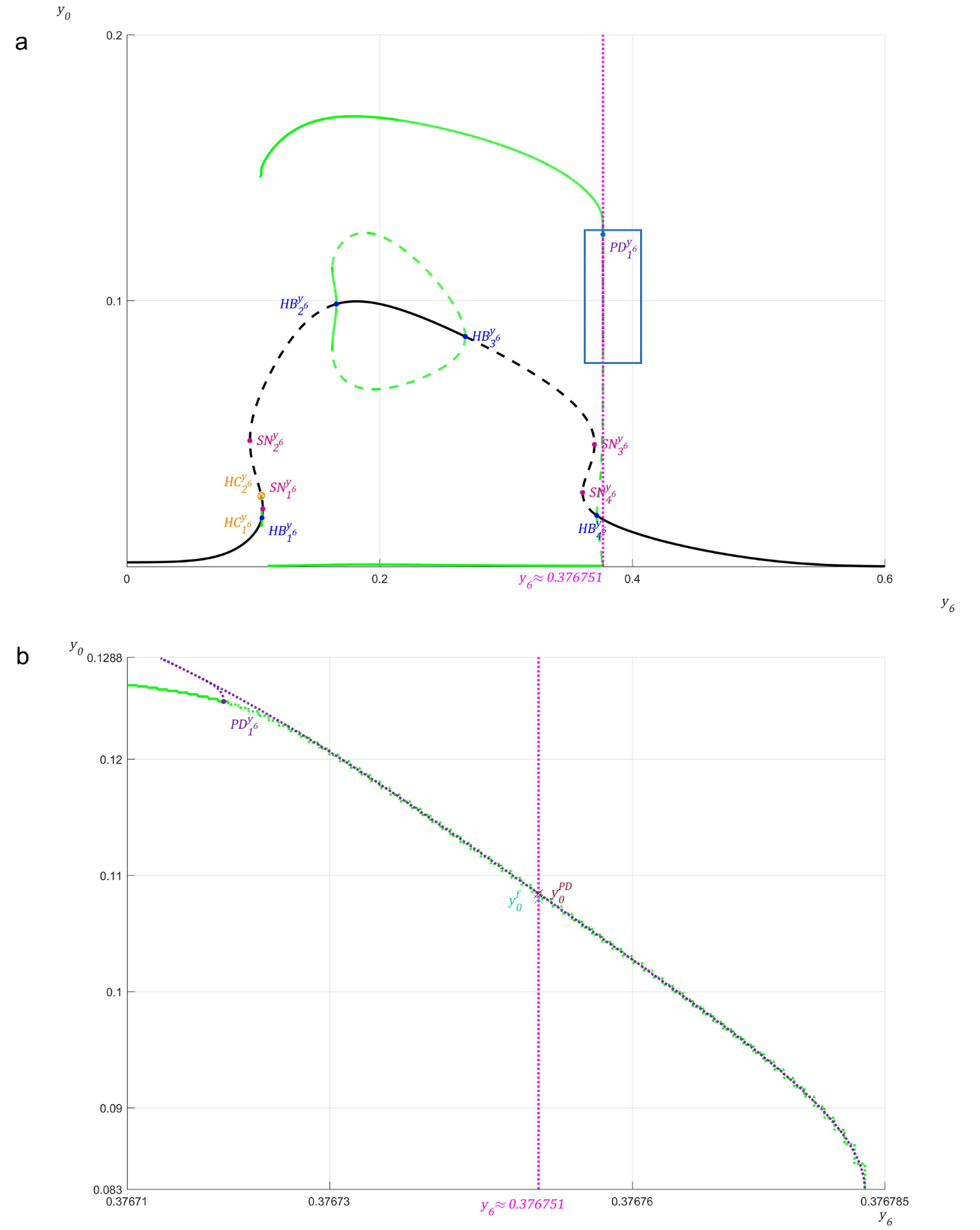

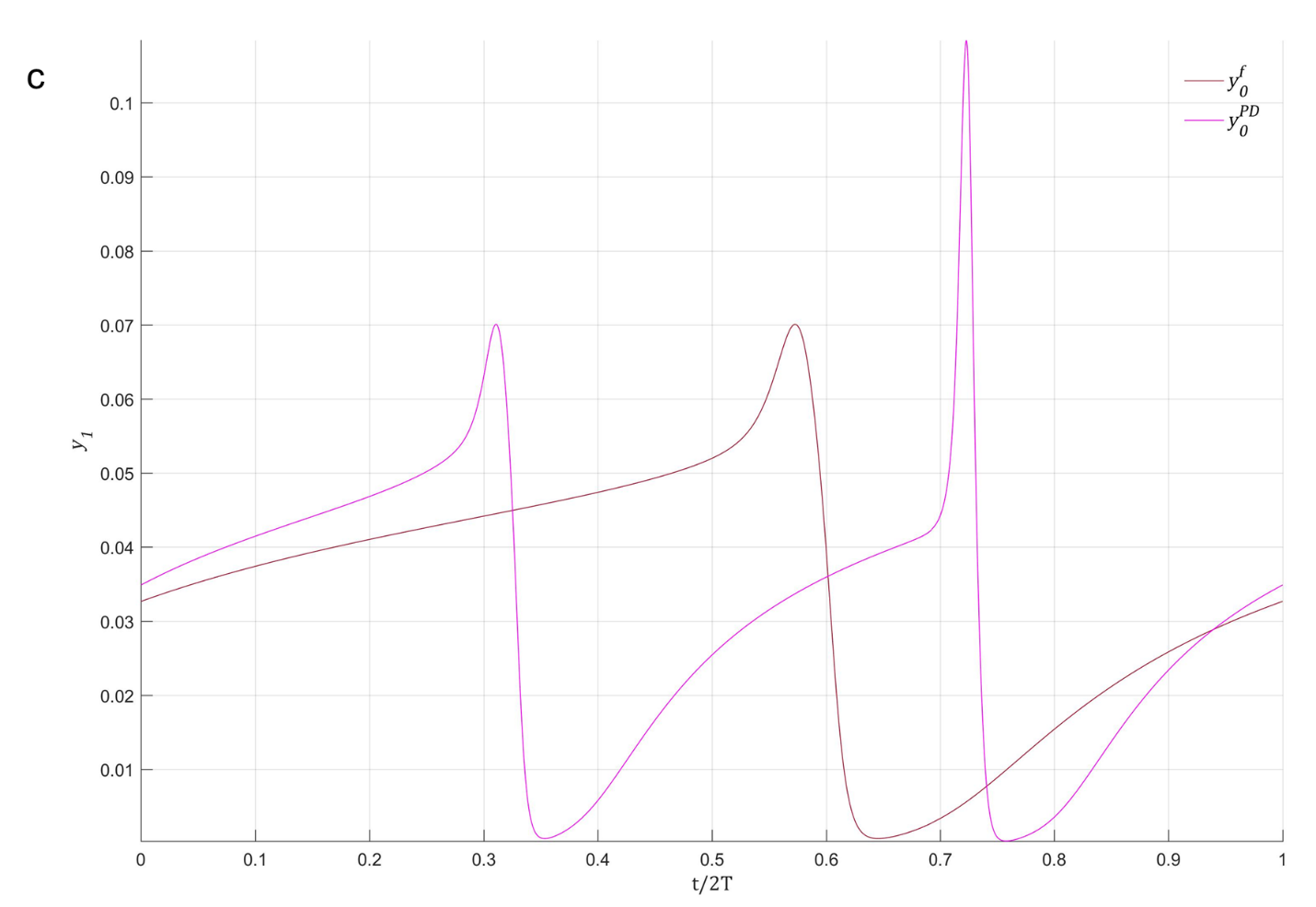


**Fig L. Bifurcation diagram for neocortical sub-system to thalamic drive: Period doubling.**

(a) Bifurcation diagram of the neocortical pyramidal PSP ($y_{0}$) versus thalamic PSP ($y_{6}$), as presented in Fig 7. Vertical line at $y_{6}\approx0.376751$ intersects with limit cycles branch and period doubling branch. (b) Zoomed in plot of the blue box in (a), depicting the intersection points of vertical at $y_{6}\approx0.376751$ at $y_{0}^{f}$ and $y_{0}^{PD}$, on the limit cycles branch and the period doubling branch, respectively. (c) Plot of $y_{1}$ vs $t/2T$ for the points $y_{0}^{f}$ and $y_{0}^{PD}$from (b).

**Appendix K: Simulated thalamic SEEG**


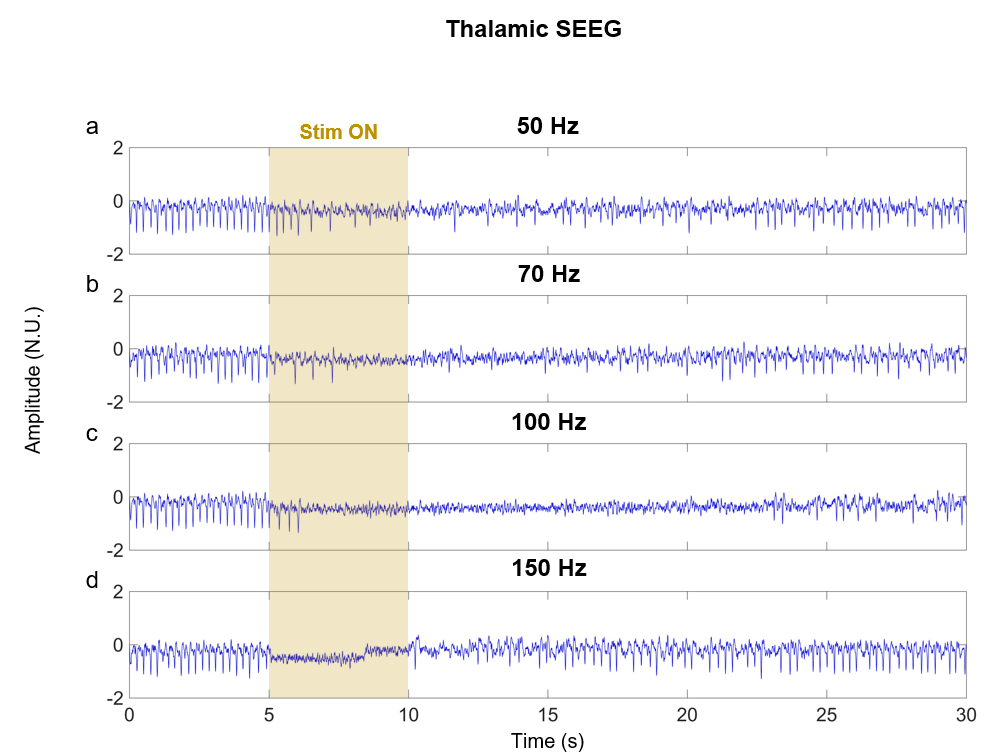


**Fig M. Simulated thalamic** **StereoElectroEncephaloGram (SEEG) during centromedian nucleus stimulation (CMS).**

SEEG with (a) 50 Hz, (b) 70 Hz, (c) 100 Hz, and (d) 150 Hz CMS at 5-10 s of the simulation.

**Appendix L: Noise analysis**

The effect of noise was quantified for each stimulation frequency by quantifying the number of interictal spikes detected in 5 s time bins of the simulated signal ($n$ = 30). Each realisation had different noise initializations.


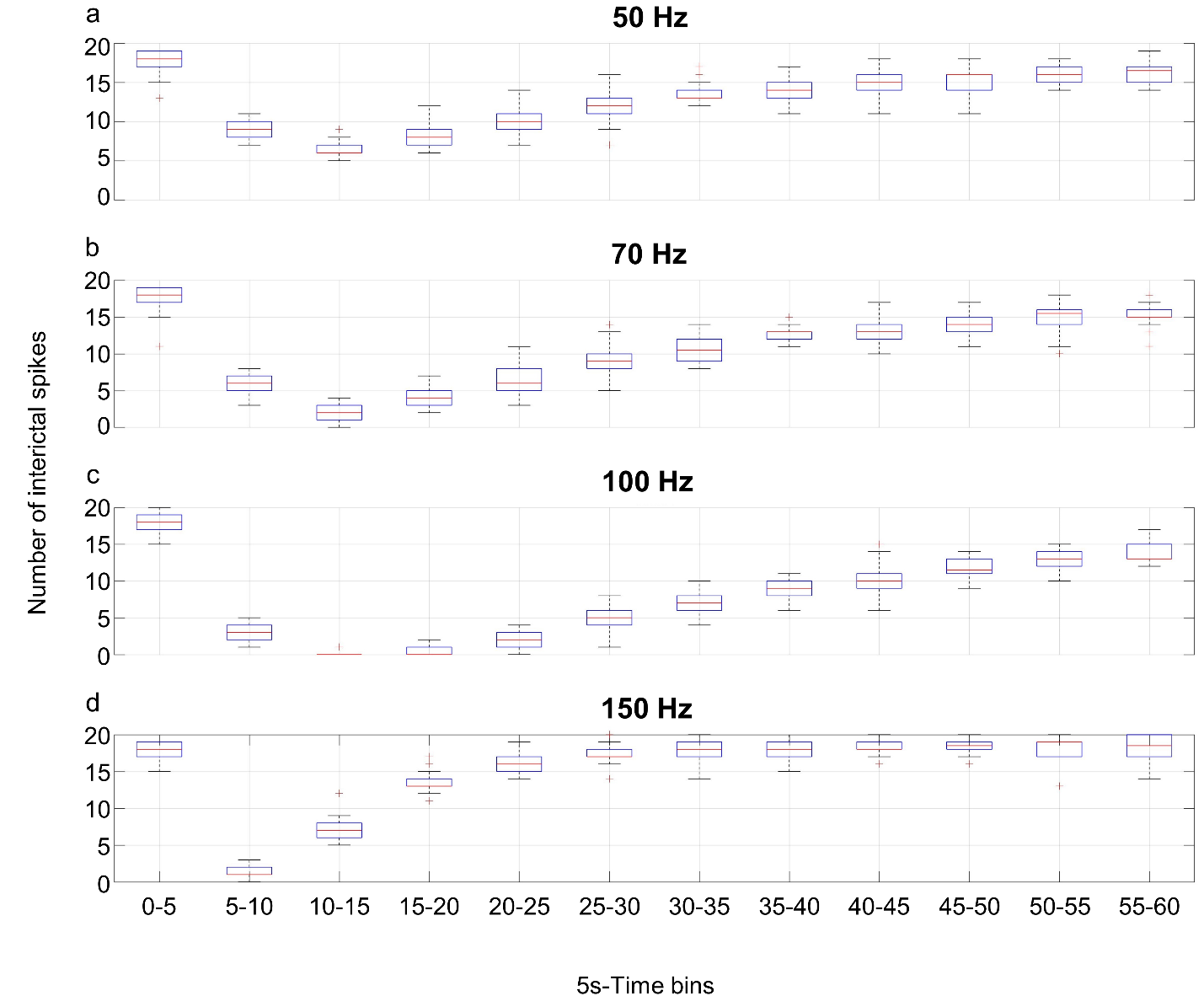


**Fig N. Effect of variation in input noise.**

Boxplot representation for the number of interictal spikes, with centromedian nucleus stimulation (CMS) at 5-10 s time bin with (a) 50 Hz, (b) 70 Hz, (c)100 Hz, and (d) 150 Hz, quantified over $n$ = 30 realizations of the neocortical signals, with different noise initializations.


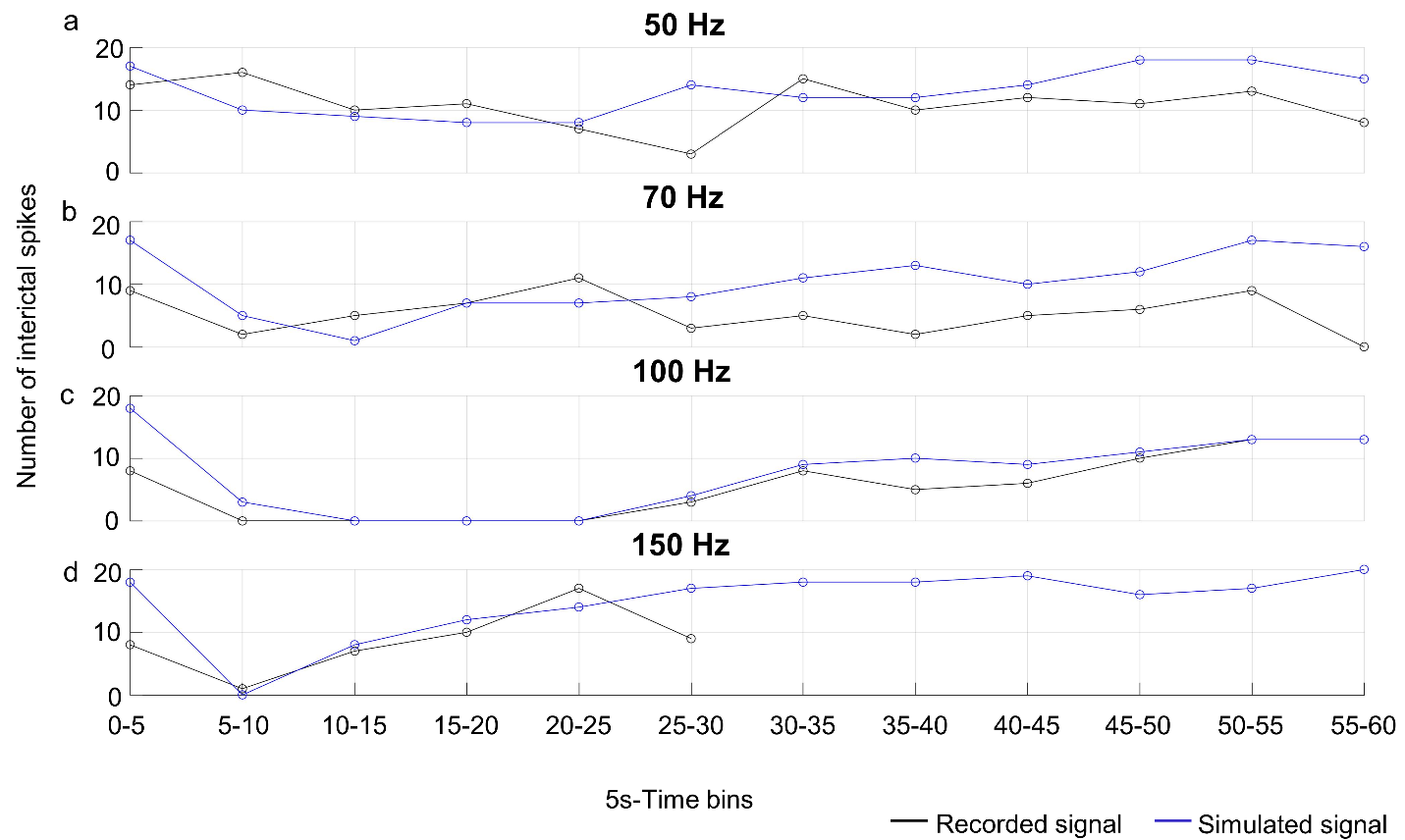


**Fig O. Number of interictal spikes in displayed signals for Fig 4.**

The interictal spike counts recorded from the recorded and simulated StereoElectroEncephaloGram signals, during centromedian nucleus stimulation (CMS) applied at (a) 50 Hz, (b) 70 Hz, (c) 100 Hz, and (d) 150 Hz in the 5-10 s time bin.

**Appendix M: Thalamic postsynaptic potential without applied mechanisms**

To examine the effect of the electrical field on the thalamic compartment, here we present the postsynaptic potentials (PSPs) simulated in this compartment. When centromedian nucleus stimulation was applied to the thalamic compartment, we observed a rise in the inhibitory-PSPs (IPSPs), with respect to their amplitude pre-stimulation. This increase in IPSPs may explain the decrease in the number of interictal spikes noted when none of the explored mechanisms were active in the model (Fig 5).


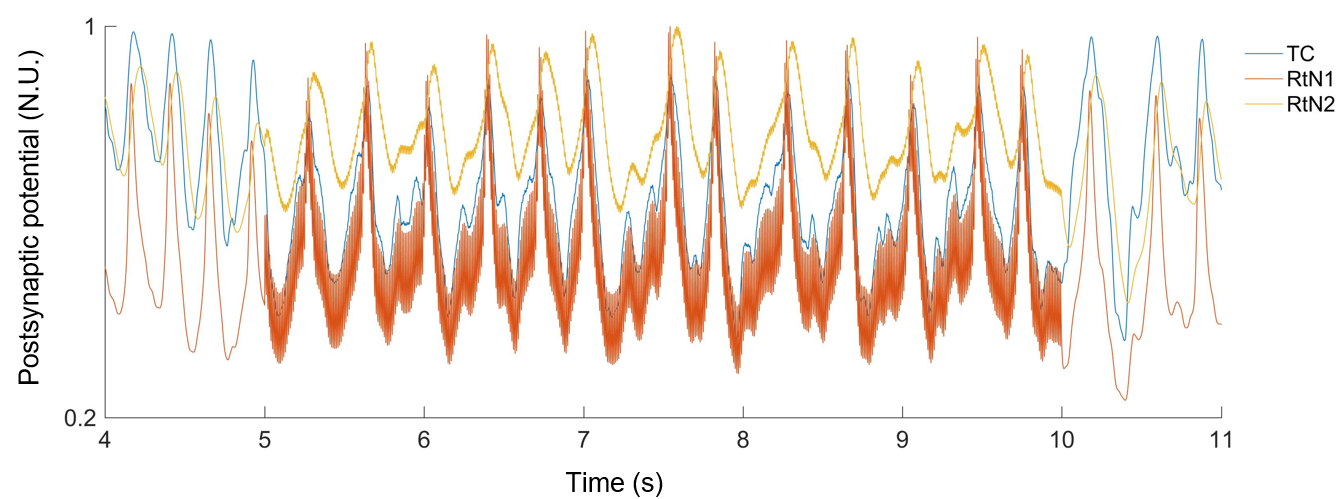


**Fig P. Simulated thalamic postsynaptic potentials (PSPs).**

Simulated PSP from the thalamocortical relay cells subpopulation (TC), fast-kinetic expressing inhibitory subpopulation (RtN1), and slow-kinetics expressing inhibitory subpopulation (RtN2) when centromedian nucleus stimulation was applied at 5-10 s of the simulation. (N.U.: normalized units)

**Appendix N****: PYR- PV-positive interneurons network**

We explored the impact of varying the connectivity parameters in the Pyr-PV network by varying 3 connectivity parameters- $C_{Pyr-PV}$, $C_{PV-Pyr}$ and $C_{PV-PV}$. For this we simulated 2100 local field potentials corresponding to different parameter configurations, where $C_{Pyr-PV}$ varied from 0 to 60 with a step size of 15, $C_{PV-Pyr}$ varied from 0 to 200 with a step size of 10, and $C_{PV-PV}$ varied from 0 to 190.95 with a step size of 10.05. The sampling rate of the recorded and simulated signals were 500 Hz.

We computed the non-linear correlation coefficient, $h^{2},$ as a brute force similarity measure for comparing the recorded and simulated signals [12]. This was done by comparing the recorded neocortical signal (signal duration = 1s) with the simulated neocortical signals (signal duration = 10s), indicated as $h_{XY}^{2}$. The recorded signal was compared against a 1s-segments of the simulated signal with a sliding window. This sliding window shifted across the simulated signal, such that the recorded signal was compared against multiple segments of the simulated signal. The window shift was set to 10 samples. This resulted in 2 sets of $5\times21\times20\times450$ data matrices.

A high $h^{2}$ value (close to 1) indicated a higher degree of similarity between the compared signals. A signal with high similarity with the recorded signal should have a high $h^{2}$ measure consistently, i.e., the maximum should be high, the minimum should be high, the mean should be high, and the standard deviation should be low. These signal features were as shown in Fig Q.

For $h_{XY}^{2}$, it was seen that a maximum ($h_{XY}^{2}=0.59$) was obtained for the parameter setting corresponding to $C_{Pyr-PV}=0$, $C_{PV-Pyr}=30$,$C_{PV-PV}=0$. The parameter settings where $C_{Pyr-PV}=0$ would be physiologically inaccurate. The next maximum for $h_{XY}^{2}=0.551$ was at $C_{Pyr-PV}=15$, $C_{PV-Pyr}=30$,$C_{PV-PV}=0$. But $h^{2}$ signal at this parameter setting showed a high standard deviation ($>0.09$). The next maximum at $h_{XY}^{2}=0.54$ was at $C_{Pyr-PV}=30$, $C_{PV-Pyr}=10$,$C_{PV-PV}=20.5$, and this may be the best parameter setting based on $h_{XY}^{2}$.


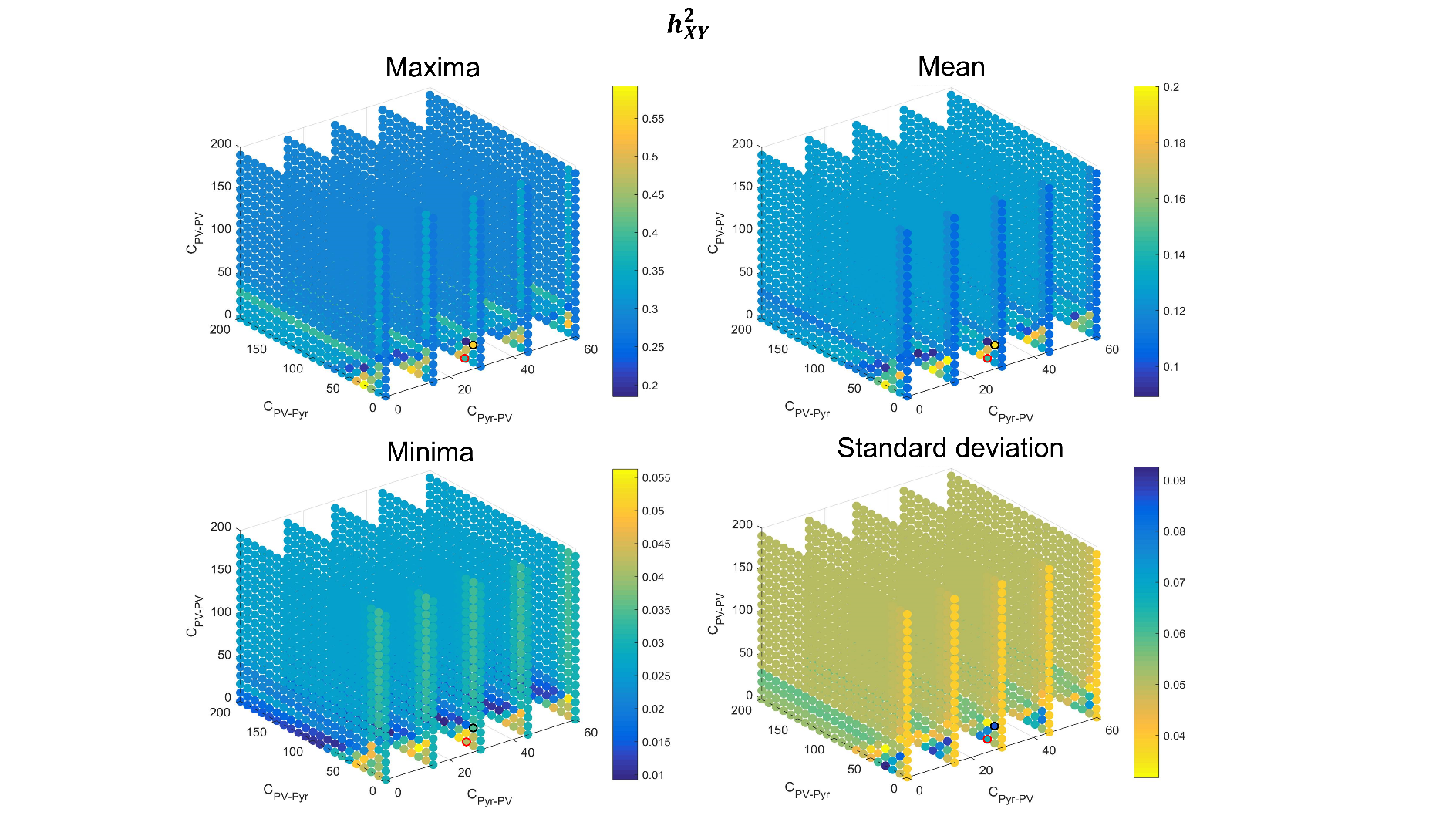


**Fig Q. Signal similarity measure:** $\boldsymbol{h}_{\boldsymbol{XY}}^{\boldsymbol{2}}\boldsymbol{.}$

The maxima, minima, mean and standard deviation of the similarity measure, $h_{XY}^{2}$, obtained when comparing the recorded neocortical StereoElectroEncephaloGram (SEEG) against the simulated neocortical SEEG. The data points corresponding to parameter sets $C_{Pyr-PV}=30$, $C_{PV-Pyr}=10$,$C_{PV-PV}=20.5$ has been circled in black (**○**), and the data point corresponding to $C_{Pyr-PV}=30$, $C_{PV-Pyr}=20$,$C_{PV-PV}=0,$has been circled in red (○).

On comparing the simulated SEEG signals corresponding to the parameter setting $C_{Pyr-PV}=30$, $C_{PV-Pyr}=10$,$C_{PV-PV}=20.5$, with the simulated SEEG signal for the parameter setting used in this study, i.e., $C_{Pyr-PV}=30$, $C_{PV-Pyr}=20$,$C_{PV-PV}=0$, it was seen that the signal morphology remained the same for these three parameter settings, as seen in Fig R.


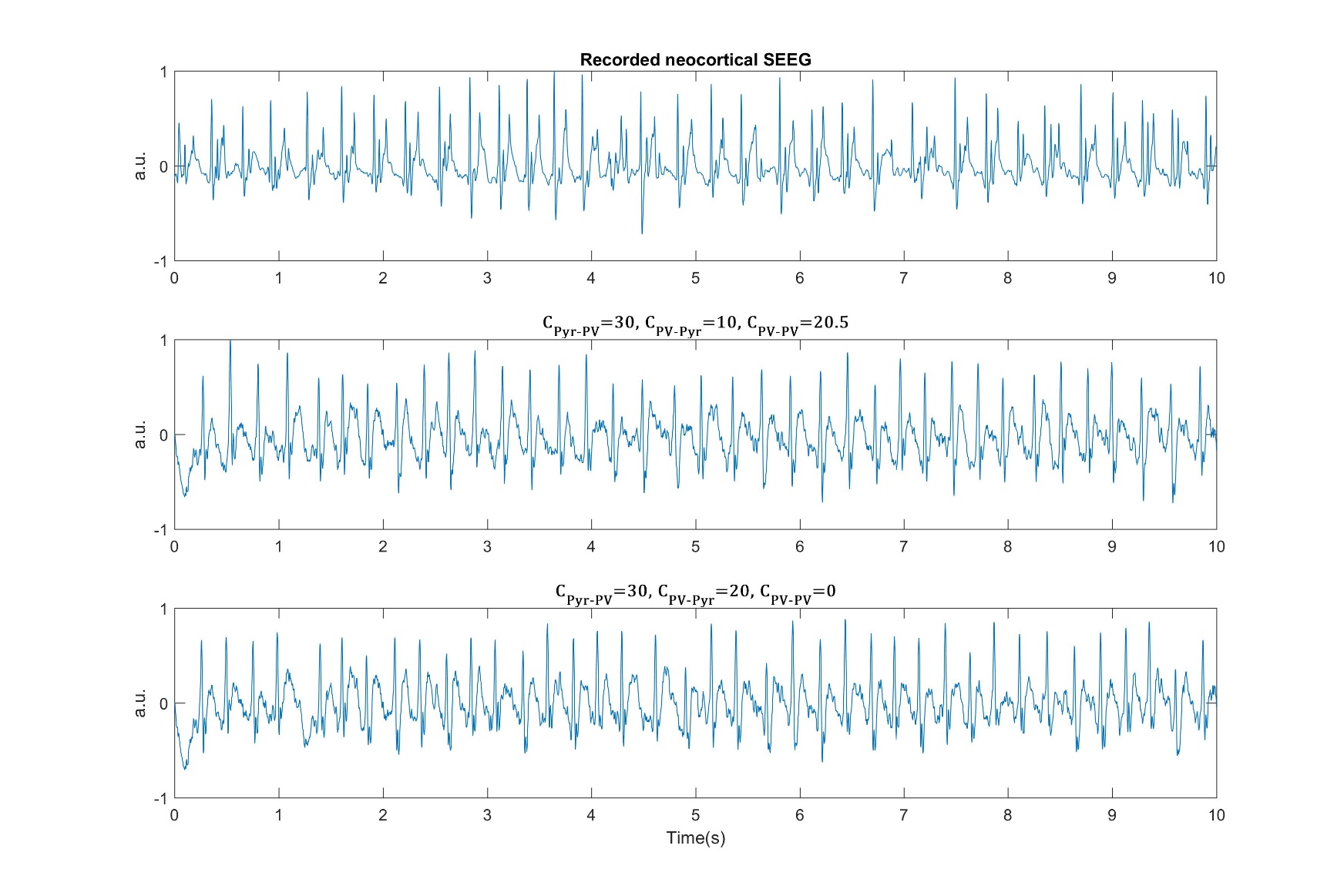


(c)

(b)

(a)

**Fig R. Neocortical StereoElectroEncephaloGram (SEEG) signal.**

(a) Recorded neocortical SEEG signal from dysplastic tissue, and the simulated neocortical SEEG for the parameter setting corresponding to (b) $C_{Pyr-PV}=30$, $C_{PV-Pyr}=20$,$C_{PV-PV}=0$, (c) $C_{Pyr-PV}=30$, $C_{PV-Pyr}=10$,$C_{PV-PV}=20.5$. (a.u.: arbitrary units)

**References**

1. Nettleton, J.S. and W.J. Spain, *Linear to Supralinear Summation of AMPA-Mediated EPSPs in Neocortical Pyramidal Neurons.* Journal of Neurophysiology, 2000. **83**(6): p. 3310-3322.

2. Xiang, Z., J.R. Huguenard, and D.A. Prince, *GABAA receptor-mediated currents in interneurons and pyramidal cells of rat visual cortex.* The Journal of Physiology, 1998. **506**(3): p. 715-730.

3. Campbell, P.W., et al., *Synaptic properties of the feedback connections from the thalamic reticular nucleus to the dorsal lateral geniculate nucleus.* J Neurophysiol, 2020. **124**(2): p. 404-417.

4. Sieber, A.R., R. Min, and T. Nevian, *Non-Hebbian long-term potentiation of inhibitory synapses in the thalamus.* J Neurosci, 2013. **33**(40): p. 15675-85.

5. Astori, S. and A. Luthi, *Synaptic plasticity at intrathalamic connections via CaV3.3 T-type Ca2+ channels and GluN2B-containing NMDA receptors.* J Neurosci, 2013. **33**(2): p. 624-30.

6. Yang, Q., et al., *Intrinsic properties of and thalamocortical inputs onto identified corticothalamic-VPM neurons.* Somatosens Mot Res, 2014. **31**(2): p. 78-93.

7. Deleuze, C. and J.R. Huguenard, *Two classes of excitatory synaptic responses in rat thalamic reticular neurons.* J Neurophysiol, 2016. **116**(3): p. 995-1011.

8. Gentet, L.J. and D. Ulrich, *Strong, reliable and precise synaptic connections between thalamic relay cells and neurones of the nucleus reticularis in juvenile rats.* J Physiol, 2003. **546**(Pt 3): p. 801-11.

9. Dilger, E.K., H.S. Shin, and W. Guido, *Requirements for synaptically evoked plateau potentials in relay cells of the dorsal lateral geniculate nucleus of the mouse.* J Physiol, 2011. **589**(Pt 4): p. 919-37.

10. Simko, J. and H. Markram, *Morphology, physiology and synaptic connectivity of local interneurons in the mouse somatosensory thalamus.* J Physiol, 2021. **599**(22): p. 5085-5101.

11. Köksal-Ersöz, E., et al., *eCOALIA: Neocortical neural mass model for simulating electroencephalographic signals.* SoftwareX, 2024. **28**: p. 101924.

12. Wendling, F., et al., *Interpretation of interdependencies in epileptic signals using a macroscopic physiological model of the EEG.* Clin Neurophysiol, 2001. **112**(7): p. 1201-18.
